# Dentition Status and Denture Use in Relation to Later-Life Health Transitions in Older Chinese Adults

**DOI:** 10.1016/j.identj.2026.109710

**Published:** 2026-07-04

**Authors:** Jin Wang, Xiaojuan Su, Ziqi Yang, Yihao Pei, Wenqiang Lu, Yiming Mao, Kan Xu, Ying Yuan

**Affiliations:** a Medical College of Jinzhou Medical University, Jinzhou, China; b Department of Geriatrics, Zhongshan Hospital (Xiamen), Fudan University, Xiamen, China; c Department of Thoracic Surgery, Suzhou Kowloon Hospital, Shanghai Jiao Tong University School of Medicine, Suzhou, China; d Department of Geriatrics, Zhongshan Hospital, Fudan University, Shanghai, China

**Keywords:** ADL disability, Ageing, Cohort study, Dementia, Denture use, Edentulism

## Abstract

**Introduction and aims:**

We examined whether baseline dentition status was associated with transitions to activities of daily living (ADL) disability, dementia, both ADL disability and dementia, and death in older Chinese adults, and whether denture use further distinguished risk within low-dentition groups.

**Methods:**

We analysed Chinese Longitudinal Healthy Longevity Survey data from 2008 to 2018. Participants were aged 65 years or older, had baseline natural tooth count data, and had no observed ADL disability or reported dementia at baseline. Dentition status was classified as at least 20 teeth, 1-19 teeth, and 0 teeth. Transition-specific discrete-time complementary log-log models were fitted within a cumulative five-state framework comprising Healthy, ADL disability only, dementia only, both ADL disability and dementia, and death.

**Results:**

A total of 10,186 participants contributed 18,688 interval-level observations. The most frequent transitions were to death (n = 5,063) and ADL disability only (n = 2,048). Compared with participants with at least 20 teeth, those with 0 teeth had higher hazards of ADL disability only (HR: 1.20, 95% CI, 1.04-1.39) and death (HR: 1.18, 95% CI, 1.06-1.31). In joint analyses, those with 0 teeth and dentures had the highest hazard of ADL disability only (HR: 1.30, 95% CI, 1.10-1.53), whereas those with 0 teeth and no dentures had the highest hazard of death (HR: 1.33, 95% CI, 1.18-1.49). Dementia-related transitions were sparse.

**Conclusion:**

Among older Chinese adults without observed ADL disability or reported dementia at baseline, edentulism was associated with less favourable later-life transitions, most consistently to ADL disability and death. Baseline dentition status may serve as a simple marker of oral functional reserve and broader later-life vulnerability.

**Clinical Relevance:**

Baseline dentition and denture status may help identify older adults who warrant broader assessment of function, nutrition, frailty, and oral rehabilitation needs.

## Introduction

Tooth loss and edentulism remain major challenges in ageing populations because they are common, cumulative, and closely tied to daily function and quality of life.^1^ Recent global burden estimates continue to show that edentulism contributes substantially to disability burden in later life.^2^ In geriatric oral health, retention of 20 or more natural teeth remains a practical benchmark for functional dentition and healthier ageing.^3^ That threshold is clinically meaningful rather than arbitrary, because cohort evidence has associated the presence of 20 or more natural teeth with more favourable survival in older adults.^4^

An expanding longitudinal literature has begun to treat oral status less as an isolated dental trait and more as a marker of broader vulnerability in old age. In the English Longitudinal Study of Ageing, total tooth loss was associated with later physical and cognitive decline.^5^ Japanese prospective studies have likewise linked tooth loss to incident functional disability, and some have suggested that dental care or rehabilitation may modify that association.^6^^,^^7^ Meta-analytic evidence also supports associations between tooth loss and later cognitive decline or dementia, although between-study heterogeneity remains.^8^

Reduced dentition, however, is unlikely to be fully captured by tooth count alone. Impaired dentition has been associated with subsequent deterioration in dietary intake, and tooth loss in older adults is linked to higher risk of malnutrition.^9^^,^^10^ Measures of oral function such as occlusal force and oral frailty also predict disability and mortality, suggesting that the relevant signal may reflect functional reserve rather than tooth number alone.^11^^,^^12^ Recent longitudinal evidence has further linked poorer subjective masticatory function with mild cognitive impairment, reinforcing the need to interpret tooth count alongside oral functional reserve.^13^ Rehabilitation may therefore matter. In Chinese cohort data, denture use has been associated with attenuation of the adverse associations of tooth loss with cognitive impairment and lower mortality.^14^^,^^15^ More recent longitudinal evidence likewise suggests slower cognitive decline among older adults with partial tooth loss who use dentures, and lower all-cause and cause-specific mortality among older adults with fewer teeth who wear dentures.^16^^,^^17^

Even so, many longitudinal studies still evaluate one outcome at a time, such as disability, cognitive impairment, or death, despite the fact that late-life health often unfolds through interrelated transitions rather than isolated endpoints.^18^^,^^19^ This is relevant to oral health because older adults without observed ADL disability or reported dementia at baseline may still differ markedly in baseline dentition status, and subsequent progression may not follow a single pathway. Some may develop ADL disability while remaining free of reported dementia, some may develop dementia, some may accumulate both ADL disability and dementia, and others may die before either nonfatal state is clearly observed. Denture use may also carry additional information beyond tooth count within low-dentition strata. We therefore used the Chinese Longitudinal Healthy Longevity Survey to examine whether baseline natural tooth count was associated with cumulative multistate progression from a survey-defined healthy baseline state to ADL disability only, dementia only, both ADL disability and dementia, and death, while also evaluating denture use as an additional baseline oral-status marker. We interpreted baseline dentition status as a clinically accessible marker of oral functional reserve and broader later-life vulnerability, and hypothesised that complete edentulism would be most clearly associated with less favourable transitions from the healthy state.

## Materials and methods

### Data source and study design

We conducted a prospective cohort study using data from the Chinese Longitudinal Healthy Longevity Survey, a nationwide longitudinal study of older adults in China.^20^ We treated the 2008 wave as baseline and followed participants through the 2011, 2014, and 2018 waves. CLHLS data were collected through structured interviews; when participants were unable to respond directly, selected information could be provided by proxy respondents according to survey procedures. The aim of the present analysis was to evaluate whether baseline dentition status could serve as a simple oral-health marker of subsequent transitions from a survey-defined healthy baseline state to ADL disability, dementia, both ADL disability and dementia, and death.

We used a cumulative multistate framework to examine progression across prespecified health states among participants without observed ADL disability or reported dementia at baseline.

### Study population

Participants were eligible if they were aged 65 years or older in 2008 and had baseline information on natural teeth count available. The age threshold of 65 years was selected a priori to align the analysis with conventional geriatric epidemiology and to define an older-adult cohort; retirement-defined eligibility was not used because retirement age in China varies by sex and occupational history. We further restricted the cohort to those in a baseline healthy state, defined as having neither observed ADL disability nor reported dementia at baseline.

The primary analytic cohort included participants who contributed at least one usable follow-up interval after baseline under the prespecified sequential follow-up rule. For the secondary joint-exposure analysis, participants additionally required baseline denture information. Because baseline denture data were complete after application of the main cohort restrictions, the joint-exposure cohort was identical in size to the primary analytic cohort.

### Exposure assessment

The primary exposure was baseline dentition status, classified into three predefined categories according to the number of natural teeth at baseline: at least 20 teeth, 1-19 teeth, and 0 teeth. These categories were selected to reflect clinically distinct levels of oral functional reserve, with complete edentulism treated as the most severe baseline oral state.

A secondary joint-exposure analysis combined baseline dentition status with baseline denture use, producing six categories: at least 20 teeth without dentures, at least 20 teeth with dentures, 1-19 teeth without dentures, 1-19 teeth with dentures, 0 teeth without dentures, and 0 teeth with dentures. In an additional internal comparison, denture use was evaluated within participants whose baseline dentition was below 20 teeth.

The tooth-count variable captured the number of natural teeth and did not provide separate information on dental implants, occlusal units, denture quality, or prosthesis fit. Baseline dentition and denture status were retained as exposure definitions to preserve temporal ordering. Follow-up tooth-count and denture status were described in supplementary analyses but were not used to redefine baseline exposure categories.

### Outcome definition and multi-state framework

The primary outcome framework comprised cumulative progression across five states: Healthy, ADL disability only, Dementia only, Both ADL disability and dementia, and Death. ADL refers to activities of daily living. ADL disability was operationalised as a binary wave-specific indicator derived from six standard ADL items. Disability was coded when at least one item indicated limitation or dependency and all six items were observed. Thus, the available CLHLS measure captured the presence or absence of any ADL disability at each wave, rather than the number of impaired ADL items or disability severity. Dementia status was derived from the CLHLS dementia item at each wave. Death was determined from the interval-specific mortality variables spanning 2008-2011, 2011-2014, and 2014-2018.

ADL disability and dementia were carried forward cumulatively once first observed, and Death was treated as the terminal absorbing state. This cumulative coding preserved temporal coherence in the multistate framework when nonfatal states were measured only at discrete survey waves. Raw wave-to-wave reversals before cumulative carry-forward coding are reported in the Supplementary Appendix. The prespecified allowed transitions were Healthy to ADL disability only, Healthy to Dementia only, Healthy to Both ADL disability and dementia, Healthy to Death, ADL disability only to Both ADL disability and dementia, ADL disability only to Death, Dementia only to Both ADL disability and dementia, Dementia only to Death, and Both ADL disability and dementia to Death. Follow-up was divided into three intervals, 2008-2011, 2011-2014, and 2014-2018, using interval-end timing because the extracted mortality variables identified death within intervals rather than exact event dates. Because nonfatal states were assessed only at follow-up waves and death was identified within intervals, the within-interval ordering of impairment onset and death could not be fully resolved. Accordingly, transitions were classified according to interval-end observed states rather than exact within-interval event sequences.

### Person-period data construction

We created interval-specific person-period datasets for the three follow-up intervals. Each participant contributed interval-specific observations only when both the state at the start of the interval and the state at the end of the interval were observable. If an interval became unusable because of missing end-of-interval state information, later intervals were not carried forward. This strict sequential rule was applied to preserve temporal coherence in the cumulative multistate framework.

Transition-specific risk sets were then constructed for each allowed transition. For a given transition, participants entered the corresponding model whenever they were in the relevant origin state at the start of an interval. The event indicator was coded as 1 when the destination state of interest was reached by the end of that interval and 0 otherwise.

### Covariates

Baseline covariates were selected a priori and included age, sex, education, marital status, residence, current smoking, current alcohol drinking, regular exercise, sleep duration, body mass index category, hypertension, diabetes, heart disease, and stroke. In a more extended specification, Parkinson disease and epilepsy were additionally included.

A prespecified conceptual framework was used to guide covariate selection and interpretation (Figure S3). Baseline dentition status was treated as a marker of cumulative oral-health compromise and oral functional reserve that may also reflect broader ageing-related vulnerability. Model 2 was designated as the principal model because it adjusted for demographic, socioeconomic, behavioural, and general-health factors measured at baseline, including age, sex, education, marital status, residence, smoking, drinking, exercise, sleep duration, and body mass index category. Model 3 additionally adjusted for hypertension, diabetes, heart disease, stroke, Parkinson disease, and epilepsy and was interpreted as a supportive extended model, because these conditions may reflect both baseline disease burden and pathways through which later-life vulnerability is expressed.

Categorical covariates were represented with explicit missing categories during model fitting, whereas descriptive tables omitted separate missing rows when this improved readability. Age was modelled using natural splines with 3 degrees of freedom in the regression analyses; the knot and boundary-knot locations are reported in the Supplementary Appendix.

### Statistical analysis

We first summarized baseline characteristics by baseline dentition status in the primary analytic cohort. We then used transition-specific discrete-time complementary log-log models, with cluster-robust standard errors at the participant level, to estimate associations across follow-up intervals.^21^

Three nested model specifications were considered. Model 1 adjusted for follow-up interval, age, and sex. Model 2 additionally adjusted for education, marital status, residence, smoking, drinking, exercise, sleep duration, and body mass index category and was designated as the principal model. Model 3 further adjusted for hypertension, diabetes, heart disease, stroke, Parkinson disease, and epilepsy and was retained as a supportive extended model.

Because dementia-related transitions were sparse, dementia-specific estimates were presented as exploratory. The main text therefore focused on the clinically most interpretable transitions from the baseline Healthy state, namely transition to ADL disability only and transition to Death. Transition to Both ADL disability and dementia was presented as a secondary result.

Supporting analyses included an included-versus-excluded baseline comparison using standardized mean differences, a joint-exposure analysis combining dentition status and denture use, a supportive three-state analysis using Healthy, Any impairment, and Death, a healthier restricted-sample analysis, age-stratified analyses with interaction testing, an internal denture comparison within participants with baseline dentition below 20 teeth, descriptive crude cumulative-proportion analyses, raw reversal counts before cumulative carry-forward coding, baseline missingness summaries, repeated tooth-count and denture-status summaries, a complete-case sensitivity analysis for Model 2 covariates, and an exploratory interaction analysis between baseline dentition status and hypertension. All analyses were conducted in R version 4.5.1.

## Results

### Participant selection and event distribution

**Fig. 1:**
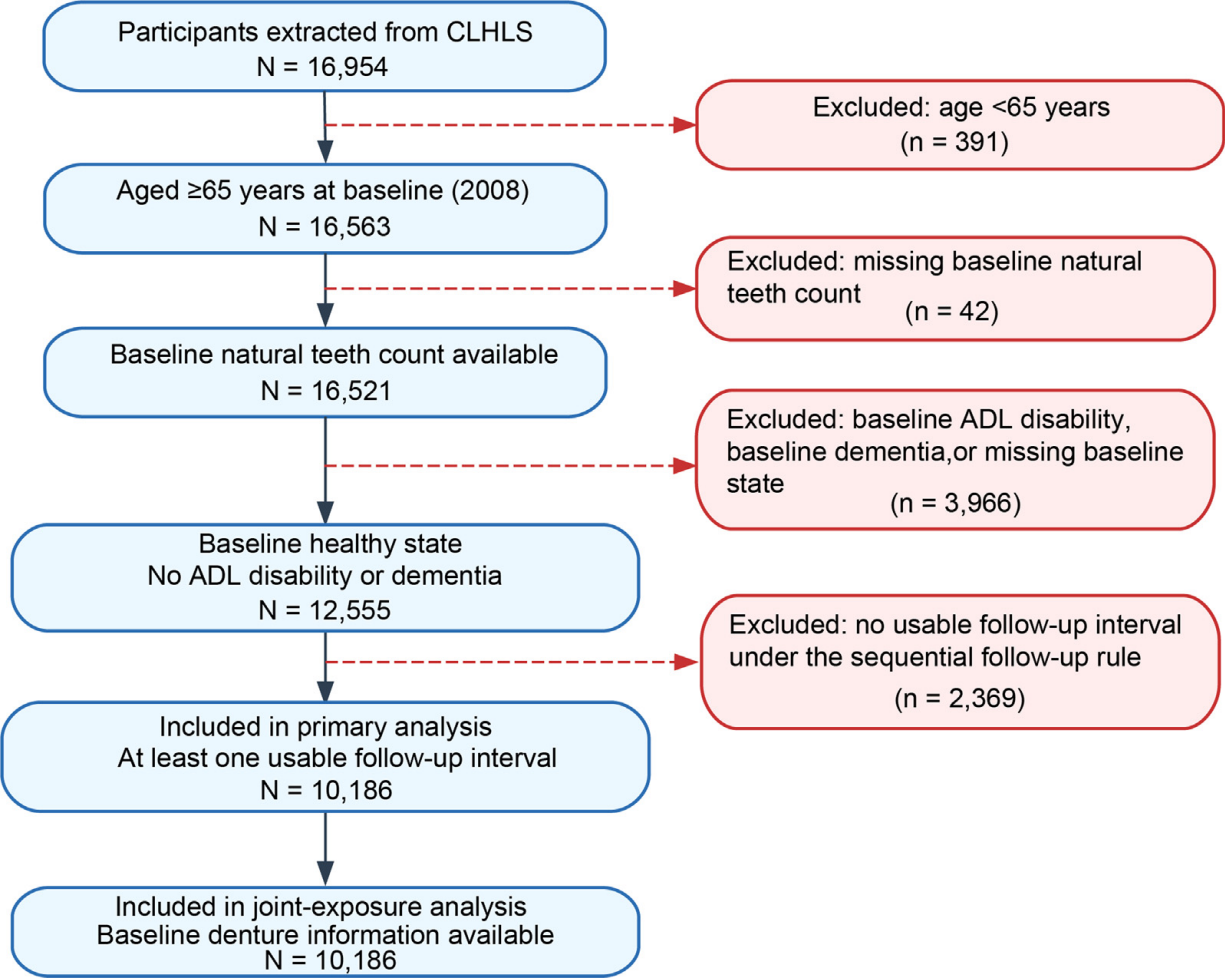
Flowchart of participant selection for the primary and joint-exposure analyses.

Figure 1 summarizes cohort derivation. A total of 16,954 participants were extracted from the CLHLS database. After restriction to those aged 65 years or older at baseline, 16,563 remained. Of these, 16,521 had baseline natural teeth information available. After exclusion of participants with baseline ADL disability, baseline dementia, or missing baseline state information, 12,555 participants were classified as baseline healthy. Following application of the strict sequential follow-up rule, 10,186 participants contributed at least one usable follow-up interval to the primary analysis, yielding 18,688 interval-level observations. The joint-exposure analysis included the same 10,186 participants and the same 18,688 interval-level observations because no additional participants were excluded for missing baseline denture information.

Across the five-state framework, the most frequent transition was Healthy to Death, with 5,063 events, followed by Healthy to ADL disability only with 2,048 events. Healthy to Both ADL disability and dementia accounted for 194 events. Dementia-related transitions were much less common, with only 85 Healthy to Dementia only events, 44 ADL disability only to Both ADL disability and dementia events, 32 Dementia only to Death events, and 18 Dementia only to Both ADL disability and dementia events. These event patterns supported a main-text emphasis on subsequent functional decline and death, while dementia-specific transitions were retained as exploratory estimates.

### Baseline characteristics

Baseline characteristics of the 10,186 participants included in the primary analytic cohort are shown in Table 1. The overall median age was 86 years. Participants with at least 20 teeth were substantially younger than those with 1-19 teeth or 0 teeth, with median ages of 73, 86, and 91 years, respectively. The edentulous group also included a higher proportion of women and lower proportions of married participants and participants with formal education.

**Table 1:** Baseline characteristics of participants included in the primary analysis by baseline dentition status.

Characteristic	Overall (N = 10,186)	≥20 teeth (N = 1,954)	1-19 teeth (N = 4,945)	0 teeth (N = 3,287)	*P* value
Sociodemographic characteristics					
Age, years	86 (76, 93)	73 (68, 81)	86 (78, 93)	91 (84, 98)	<.001
Female sex	5,436 (53%)	804 (41%)	2,662 (54%)	1,970 (60%)	<.001
**Education, years**					<.001
0	6,099 (60%)	754 (39%)	3,071 (62%)	2,274 (69%)	
1-6	3,034 (30%)	809 (41%)	1,432 (29%)	793 (24%)	
≥7	1,049 (10%)	390 (20%)	439 (8.9%)	220 (6.7%)	
Missing	4 (<0.1%)	1 (<0.1%)	3 (<0.1%)	0 (0%)	
Married	3,649 (36%)	1,219 (62%)	1,617 (33%)	813 (25%)	<.001
City/Town residence	3,545 (35%)	772 (40%)	1,673 (34%)	1,100 (33%)	<.001

Lifestyle and anthropometric factors					
Current smoking	2,085 (20%)	549 (28%)	920 (19%)	616 (19%)	<.001
Current drinking	2,017 (20%)	479 (25%)	958 (19%)	580 (18%)	<.001
Regular exercise	3,144 (31%)	810 (41%)	1,432 (29%)	902 (27%)	<.001
**Sleep duration**					<.001
<7 h	2,610 (26%)	503 (26%)	1,318 (27%)	789 (24%)	
7-9 h	4,889 (48%)	1,089 (56%)	2,382 (48%)	1,418 (43%)	
>9 h	2,661 (26%)	362 (19%)	1,232 (25%)	1,067 (32%)	
Missing	26 (0.3%)	0 (0%)	13 (0.3%)	13 (0.4%)	
**Body mass index, kg/m²**					<.001
<18.5	3,101 (30%)	345 (18%)	1,575 (32%)	1,181 (36%)	
18.5-23.9	5,556 (55%)	1,131 (58%)	2,746 (56%)	1,679 (51%)	
24.0-27.9	1,166 (11%)	380 (19%)	487 (9.8%)	299 (9.1%)	
≥28	276 (2.7%)	88 (4.5%)	103 (2.1%)	85 (2.6%)	
Missing	87 (0.9%)	10 (0.5%)	34 (0.7%)	43 (1.3%)	

Oral status					
Denture use	2,839 (28%)	280 (14%)	1,004 (20%)	1,555 (47%)	<.001

Comorbidities					
Hypertension	1,974 (19%)	444 (23%)	972 (20%)	558 (17%)	<.001
Diabetes	231 (2.3%)	65 (3.3%)	103 (2.1%)	63 (1.9%)	.008
Heart disease	824 (8.1%)	196 (10%)	387 (7.8%)	241 (7.3%)	.007
Stroke	423 (4.2%)	108 (5.5%)	191 (3.9%)	124 (3.8%)	.011

Values are median (Q1, Q3) for age and n (%) for categorical variables. Percentages are column percentages. *P* values were calculated using the Kruskal-Wallis test for age and Pearson chi-square tests for categorical variables. Missing categories were retained during model fitting but are shown in the table only for selected variables to improve readability.

Clear gradients were also present for several health-related characteristics. Participants with 0 teeth more often had low body mass index and were more likely to report sleep duration longer than 9 hours, whereas denture use was most common in the edentulous group, intermediate in the 1–19-teeth group, and least common among those with at least 20 teeth. Overall, poorer baseline dentition status clustered with older age and a less favourable general profile.

### Main transition-specific associations from the baseline healthy state

**Fig. 2:**
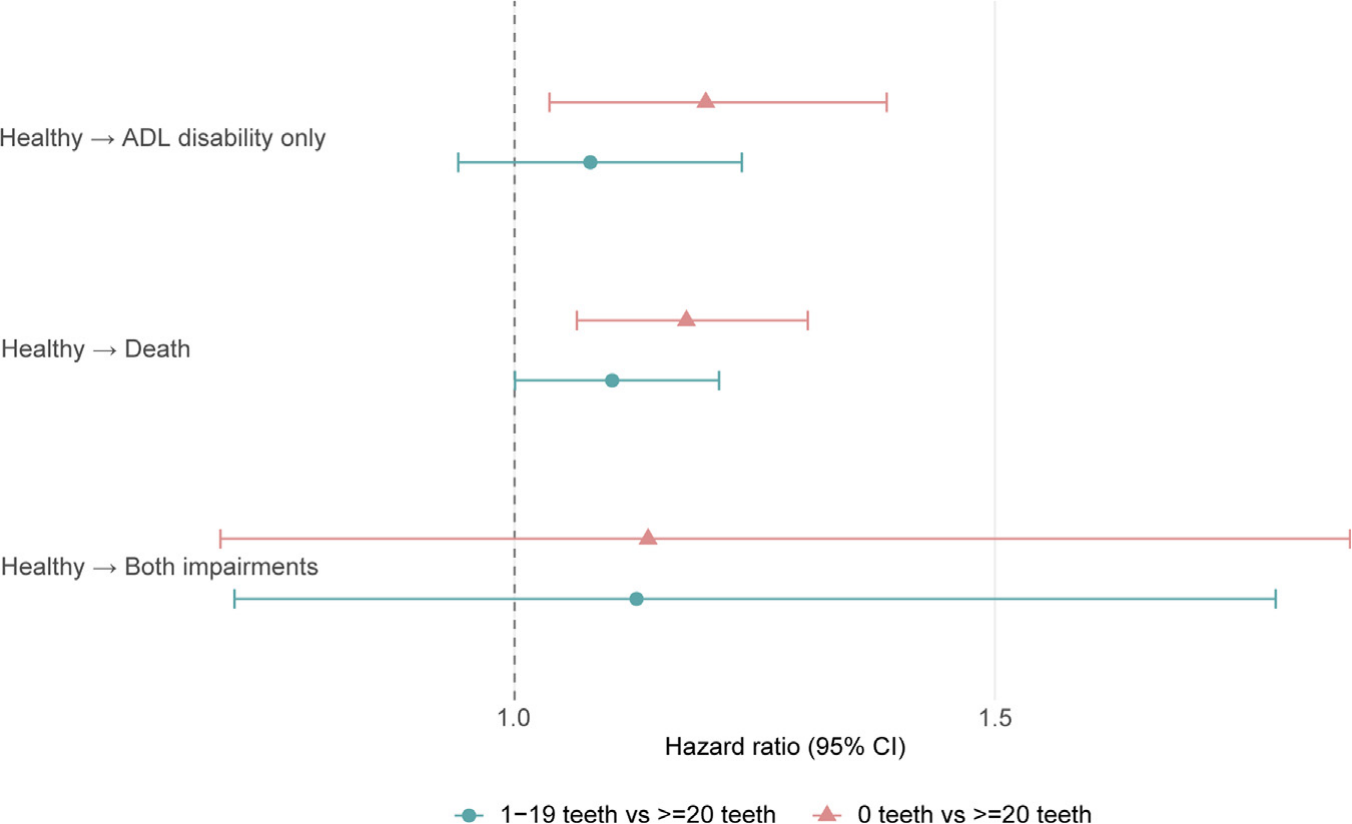
Baseline dentition status and subsequent transitions from a healthy baseline state. Hazard ratios with 95% confidence intervals were estimated from Model 2. The figure shows selected transitions from the baseline Healthy state. Participants had no observed ADL disability or reported dementia at baseline, and those with at least 20 teeth served as the reference group.

Table 2 and Figure 2 present the principal transition-specific estimates from Model 2. The clearest association was observed for baseline edentulism. Compared with participants with at least 20 teeth, those who were edentulous at baseline had a higher hazard of transition from Healthy to ADL disability only, with an adjusted hazard ratio of 1.20 (95% CI, 1.04-1.39). By contrast, the corresponding estimate for participants with 1-19 teeth was 1.08 (95% CI, 0.94-1.24), indicating no clear elevation after adjustment.

**Table 2:** Association of baseline dentition status with selected transition-specific risks in the primary multi-state analysis.

Transition	Comparison	Events	Adjusted HR (95% CI)	*P* value
Healthy to ADL disability only	1-19 teeth vs ≥20 teeth	2,048	1.08 (0.94-1.24)	.275
	0 teeth vs ≥20 teeth		1.20 (1.04-1.39)	.015
Healthy to Death	1-19 teeth vs ≥20 teeth	5,063	1.10 (1.00-1.21)	.048
	0 teeth vs ≥20 teeth		1.18 (1.06-1.31)	.002
Healthy to Both ADL disability and dementia	1-19 teeth vs ≥20 teeth	194	1.13 (0.71-1.79)	.613
	0 teeth vs ≥20 teeth		1.14 (0.69-1.87)	.606

Exponentiated coefficients from discrete-time complementary log-log models are reported as hazard ratios. Estimates are from Model 2, adjusted for follow-up interval, age modelled with a natural spline with 3 degrees of freedom, sex, education, marital status, residence, smoking, drinking, exercise, sleep duration, and body mass index category. The Healthy to Both ADL disability and dementia transition is shown as a secondary result because the number of events was limited and the estimates were imprecise.

A similar pattern was observed for transition from Healthy to Death. Compared with participants with at least 20 teeth, the adjusted hazard ratio was 1.18 (95% CI, 1.06-1.31) for those with 0 teeth and 1.10 (95% CI, 1.00-1.21) for those with 1-19 teeth. Thus, complete edentulism showed the most consistent association with both subsequent functional decline and death, whereas the pattern for partial dentition was weaker.

Transition from Healthy to Both ADL disability and dementia was directionally similar but imprecise. The adjusted hazard ratio was 1.13 (95% CI, 0.71-1.79) for participants with 1-19 teeth and 1.14 (95% CI, 0.69-1.87) for those with 0 teeth. Given the limited number of events, this transition was treated as a secondary result.

Overall, the most consistent associations were seen for transitions from the healthy state to ADL disability only and to death, particularly among participants who were already edentulous at baseline.

### Secondary and supportive analyses

Baseline selection into the primary analytic cohort appeared selective. Compared with participants retained in the primary analysis, those excluded before entry were older and generally had less favourable baseline profiles, with a mean age of 91.55 years versus 84.97 years and a standardized mean difference of 0.605 for age. They were also more often unmarried, less physically active, and more likely to be edentulous at baseline (Table S1). Full five-state results confirmed that dementia-specific transitions were sparse, with several later dementia-related estimates unstable or not estimable (Table S2).

Joint dentition-denture analyses suggested that denture status added prognostic separation within low-dentition categories. In the joint-exposure analysis, participants with 0 teeth and dentures had the highest adjusted hazard of transition from Healthy to ADL disability only, with a hazard ratio of 1.30 (95% CI, 1.10-1.53) relative to participants with at least 20 teeth and no dentures. For transition from Healthy to Death, the highest hazard was observed among participants with 0 teeth and no dentures, with a hazard ratio of 1.33 (95% CI, 1.18-1.49), whereas the corresponding estimate for those with 0 teeth and dentures was 1.06 (95% CI, 0.94-1.19). Results from the supportive three-state analysis were concordant with the primary findings (Table S3 and Figure S1; Table S4).

The overall pattern for baseline edentulism also remained stable across additional supporting analyses. In the healthier restricted sample, baseline edentulism remained associated with higher hazards of transition from Healthy to ADL disability only (HR: 1.27, 95% CI, 1.05-1.54) and from Healthy to Death (HR: 1.19, 95% CI, 1.05-1.35). Age-stratified analyses did not show meaningful evidence of effect modification, with interaction *P* values of .671 for Healthy to ADL disability only and 0.937 for Healthy to Death. Among participants with baseline dentition below 20 teeth, denture use was associated with lower subsequent death hazard in the internal comparison (HR: 0.77, 95% CI, 0.72-0.83), whereas the corresponding estimate for Healthy to ADL disability only was 1.12 (95% CI, 1.00-1.26). Descriptive crude cumulative-proportion curves showed the same ordering across dentition groups throughout follow-up (Tables S5-S7; Figure S2).

Additional analyses were consistent with the main interpretation and provided context for outcome coding, missing data, and baseline oral-status measures. Raw wave-to-wave reversals before cumulative carry-forward coding occurred mainly for ADL disability in later intervals and were uncommon for dementia. Baseline covariate missingness was low in the primary analytic cohort, and complete-case Model 2 estimates were consistent with the main analysis. Interval-end distributions among intervals starting from Healthy showed that Death and ADL disability accounted for most observed non-Healthy transitions, whereas Dementia only and Both ADL disability and dementia remained uncommon across dentition groups. Repeated survey-reported tooth-count and denture status provided descriptive context for interpreting baseline oral status as a baseline marker rather than a complete longitudinal oral-health record (Tables S8–S14; Figure S4). Exploratory interaction tests did not show a clear interaction pattern between baseline dentition status and hypertension (Table S15).

## Discussion

In this prospective multistate analysis of older Chinese adults without observed ADL disability or reported dementia at baseline, the clearest associations were observed among participants who were already edentulous at study entry. Compared with those with at least 20 teeth, baseline edentulism was associated with higher subsequent hazards of transition from the Healthy state to ADL disability only and from the Healthy state to Death, whereas the corresponding estimates for participants with 1-19 teeth were smaller and less consistent. Dementia-related transitions were uncommon and were presented as exploratory estimates. In the joint-exposure analysis, those with 0 teeth and dentures had the highest hazard of transition to ADL disability only, whereas those with 0 teeth and no dentures had the highest hazard of transition to Death. This pattern supports the interpretation of baseline dentition status as a marker of oral functional reserve and broader later-life vulnerability, with the most stable information concentrated in transitions involving ADL disability and death.

These findings are more closely aligned with the longitudinal literature linking severe tooth loss to later functional decline and survival. In the English Longitudinal Study of Ageing, total tooth loss was associated with subsequent physical and cognitive decline.^5^ Japanese cohort studies likewise reported higher risks of incident functional disability among older adults with worse dental status, including analyses that considered receipt of dental care and propensity-score-based adjustment.^6^^,^^7^ In Chinese older adults, fewer natural teeth and lack of dentures have also been linked to higher all-cause mortality.^15^^,^^17^ Recent cohort evidence further suggests that edentulism may combine with cardiometabolic vulnerability, such as hypertension, to identify older adults with higher mortality risk.^22^ Read together, those studies suggest that severe tooth loss may be most informative when the outcome concerns later-life functioning or survival, which is also where the results in our analysis were most stable.

One plausible explanation is that complete tooth loss captures a stage of oral ageing that carries broader implications than partial reduction in tooth count alone. Systematic reviews have shown that better tooth retention in later life is associated with better health and quality of life.^3^ A recent review of masticatory function further indicated that reduced chewing capacity predicts several adverse health outcomes in older adults.^23^ Prospective cohort data have linked lower occlusal force to incident functional disability,^11^ and oral frailty has been associated with subsequent physical frailty, disability, and mortality.^12^ More recent reviews have extended that picture by showing that oral frailty indicators cluster with major adverse ageing outcomes, including functional disability and death.^24^^,^^25^ In Chinese cohort data, dentition status and denture use have also been associated with frailty development.^26^ Viewed in this context, severe tooth loss appeared to carry more information for some later-life pathways than for others. Among participants without observed ADL disability or reported dementia at baseline, edentulism was more clearly associated with transitions to ADL disability and death than with the dementia-related pathways captured in the same framework.

The denture findings also warrant cautious interpretation, but they still provide clinically relevant information. In Chinese older adults, denture use has previously been associated with lower cognitive impairment among people with tooth loss,^14^ and a more recent prospective study reported slower cognitive decline among older adults with partial tooth loss who used dentures.^16^ At the same time, the broader literature suggests a more complex interpretation than a simple restorative pathway. Meta-analytic work continues to link tooth loss with later cognitive decline and dementia overall,^8^ and prospective Japanese studies have also reported higher dementia risk with more severe tooth loss.^27^^,^^28^ In that context, denture use in our cohort is unlikely to represent a single biological mechanism. It may reflect oral rehabilitation, adaptation to tooth loss, healthcare engagement, self-care capacity, and survival into a stage at which prosthodontic treatment is even feasible. That mixed meaning probably explains why denture status separated the death transition more clearly than the ADL-only transition and why the joint-exposure pattern was not identical across outcomes.

Intervention-oriented and function-oriented research points in the same direction. Replacing missing teeth does not reliably improve diet when prosthetic treatment is delivered in isolation, whereas benefit appears more plausible when oral rehabilitation is paired with dietary support.^29^ Older adults with tooth loss are also at increased risk of malnutrition, which reinforces the idea that oral rehabilitation is only one part of a wider nutritional and behavioral picture.^10^ Neurophysiologic work further suggests that prosthodontic rehabilitation may influence regional brain activity and sensory-motor processing.^30^^,^^31^ These observations support a broader interpretation of denture status as a marker of oral rehabilitation, nutritional context, and care engagement, rather than as a simple indicator of prosthetic replacement alone.

The dementia-related results should be interpreted in light of event sparsity and survey-based ascertainment. Prior cohort studies and meta-analyses continue to support associations between tooth loss and later cognitive decline or dementia.^8^^,^^27^^,^^28^ In our data, however, there were only 85 Healthy to Dementia only events overall, followed by even fewer later dementia-related transitions. Dementia may also be underdetected in community surveys and routine care,^32^ and some participants classified as having no reported dementia at baseline may have had unrecognised cognitive impairment. In a very old cohort observed at relatively wide survey intervals, cognitive decline before death may also have gone unobserved as a separate dementia state; this pattern is consistent with the broader concern that selective survival and competing mortality can shape observed cognitive-outcome associations in older cohorts.^19^^,^^33^ These features make the dementia-specific estimates less stable than the estimates for ADL disability and death.

A more substantive contribution of this study lies in the way the outcome was structured. Oral health is increasingly framed as part of healthy ageing rather than as a domain separate from general health.^34^^,^^35^ In parallel, multistate approaches in ageing research have shown that transitions among intermediate health states and death can reveal patterns that are obscured when all adverse outcomes are collapsed into a single endpoint.^19^ Our findings fit that logic. Baseline dentition status was not equally informative for every downstream path from the healthy state. Instead, the clearest separation appeared in transitions to ADL disability only and to death, whereas the dementia-related transitions contributed much less stable information empirically. That distinction is one of the most meaningful contributions of the present analysis.

This study also has methodological strengths. Because transitions were observed across survey intervals rather than at exact event times, the person-period discrete-time framework was appropriate for the structure of the data.^21^ Beginning from a baseline state without observed ADL disability or reported dementia provided a clear survey-based temporal anchor. The use of a conceptual framework clarified the role of Model 2 as the principal model and Model 3 as a supportive extended specification. In addition, the broadly similar ordering seen in the primary model, the supportive three-state model, and the healthier restricted sample reduces the likelihood that the main findings were driven entirely by one modelling choice or by heavy baseline comorbidity.

Several limitations should be considered alongside those strengths. Selection before entry into the primary analytic cohort was substantial. Participants excluded before the primary analysis were older, frailer, and more often edentulous, indicating selective entry into the analytic sample from the outset. In ageing research, such selection can distort observed associations because survival and continued participation are themselves patterned by health status.^36^ More generally, attrition in cohort studies can bias effect estimates when follow-up loss is related to both baseline characteristics and subsequent outcomes.^37^ ADL disability was analysed as a binary survey-based measure; therefore, we could not distinguish mild from severe disability or examine improvement in disability severity between waves. Tooth count and denture use in CLHLS were obtained from structured survey questions rather than clinical oral examinations, so some exposure misclassification is unavoidable. We also lacked information on caries history, periodontal status, oral hygiene, occlusal support, masticatory performance, nutritional status, denture quality, implant-supported function, dental-care access and trajectories, and reasons for tooth loss. Baseline dentition and denture status were used to preserve temporal ordering, but repeated survey data showed that oral status could change or become unobserved during follow-up; baseline oral status should therefore be interpreted as a baseline marker rather than a complete longitudinal record of oral rehabilitation or subsequent tooth loss. Because nonfatal states were only observed at follow-up waves whereas death was identified within intervals, the exact within-interval ordering of impairment onset and death could not be determined. Some participants who died during an interval may have developed ADL disability or dementia before death, but those intermediate states could not always be observed separately. This limitation is particularly relevant to dementia-related pathways, which were sparse and more dependent on survey timing.

Clinically, these findings suggest that severe tooth loss may help identify older adults who warrant broader assessment of function, nutrition, frailty, oral rehabilitation needs, and access to care. Baseline dentition status appeared most informative for transitions involving ADL disability and death, rather than for all later-life trajectories to the same degree. Within low-dentition strata, denture status provided additional separation, although its meaning is likely mixed and may reflect oral rehabilitation, care access, treatment-seeking capacity, and selective survival. This interpretation is compatible with earlier work showing that retention of 20 or more natural teeth is associated with more favourable survival in older populations.^4^

Future studies should build on that distinction rather than flatten it. Repeated measurement of tooth count, denture use, chewing ability, and periodontal status would help separate structural tooth loss from functional oral capacity. Designs that address informative attrition and competing death more explicitly would also strengthen inference in the oldest-old. More broadly, our findings suggest that late-life oral-health studies may be most informative when they examine multiple health transitions jointly, instead of assuming that one baseline oral measure will show the same relationship with every downstream outcome.

## Conclusion

In this prospective cohort study of older adults without observed ADL disability or reported dementia at baseline, complete edentulism was associated with less favourable subsequent transitions, most consistently to ADL disability and death. Within low-dentition groups, denture status provided additional risk stratification, with different patterns for functional decline and mortality transitions. Baseline dentition status may serve as a clinically accessible marker of oral functional reserve and broader later-life vulnerability in very old adults.

## Author contributions

Jin Wang: Conceptualization, Methodology, Investigation, Data curation, Validation, Writing – original draft, Writing – review and editing. Xiaojuan Su: Conceptualization, Methodology, Investigation, Data curation, Validation, Writing – original draft, Writing – review and editing. Ziqi Yang: Methodology, Software, Formal analysis, Data curation, Visualization, Validation, Writing – original draft, Writing – review and editing. Yihao Pei: Investigation, Data curation, Formal analysis, Visualization, Validation, Writing – review and editing. Wenqiang Lu: Conceptualization, Methodology, Formal analysis, Investigation, Data curation, Validation, Writing – original draft, Writing – review and editing. Yiming Mao: Conceptualization, Methodology, Resources, Supervision, Project administration, Funding acquisition, Validation, Writing – review and editing. Ying Yuan: Conceptualization, Methodology, Resources, Supervision, Project administration, Writing – review and editing. Kan Xu: Conceptualization, Methodology, Resources, Supervision, Project administration, Validation, Writing – review and editing. All authors have read and approved the final version of the manuscript.

## Institutional review board statement

The Chinese Longitudinal Healthy Longevity Survey was approved by the Ethics Committee of Peking University (IRB00001052-13074). Written informed consent was obtained from all participants or their legal representatives. The present study was a secondary analysis of de-identified CLHLS data.

## Informed consent statement

Written informed consent was obtained from all participants in the Chinese Longitudinal Healthy Longevity Survey. When a participant was unable to provide consent directly, consent was obtained from a proxy respondent in accordance with the CLHLS study protocol. This secondary analysis used de-identified data.

## Funding

This work was supported by the Education Science “Fourteenth Five-Year Plan” Project of Liaoning Province, China [Grant Number JG25DB179] and the Cultivation Program of Suzhou Kowloon Hospital [Grant Number SZJL202403].

## Conflict of interest

None disclosed.

## Appendix Supplementary materials

Image, application 1

Image, application 2

Image, application 3

Image, application 4

Image, application 5

## Footnotes

Supplementary material associated with this article can be found in the online version at doi:10.1016/j.identj.2026.109710.
