# Supplementary figures and images for: Dentition Status and Denture Use in Relation to Later-Life Health Transitions in Older Chinese Adults
Source: Int Dent J. 2026 Jul 4;76(5):109710. doi: 10.1016/j.identj.2026.109710 (PMC13355603; doi:10.1016/j.identj.2026.109710)

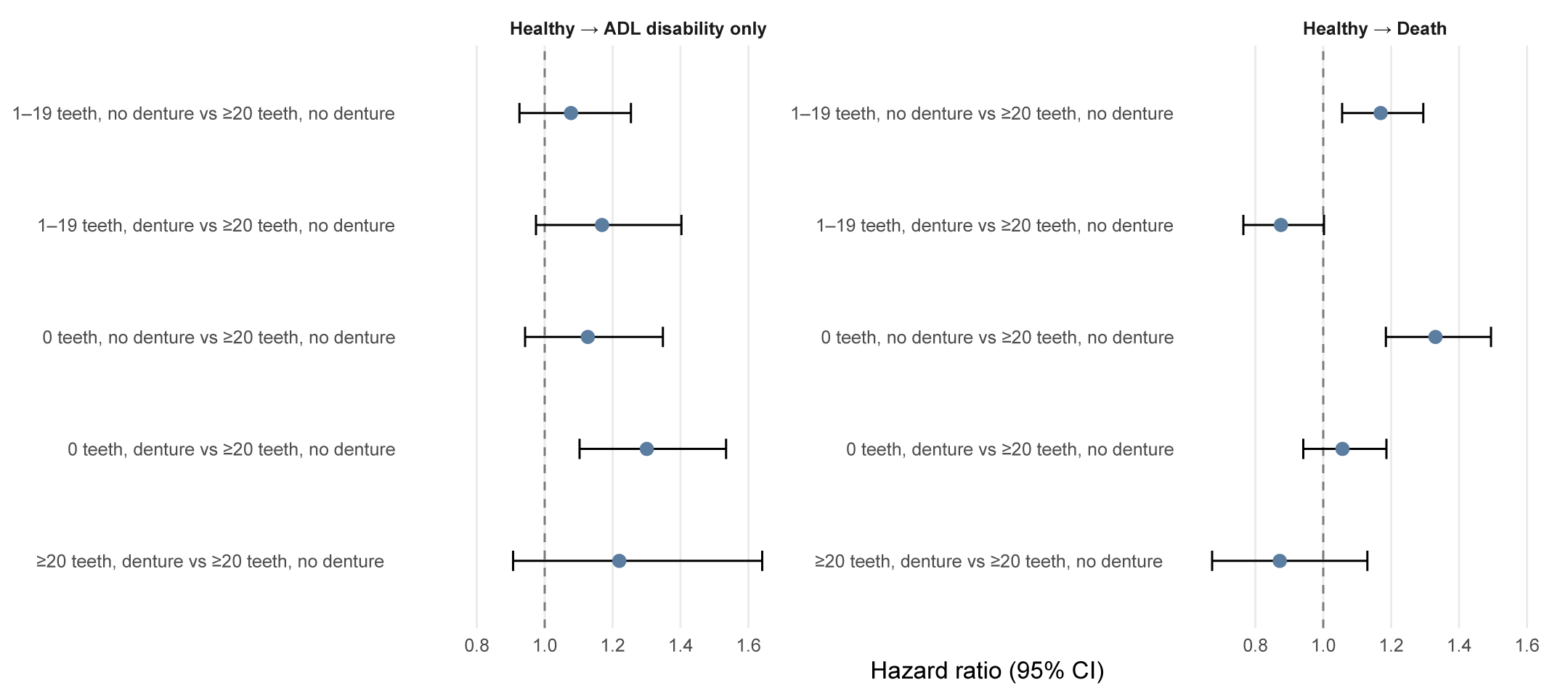

Supplement: Supplementary file 1 [file mmc1.zip › Figure S1.tif]

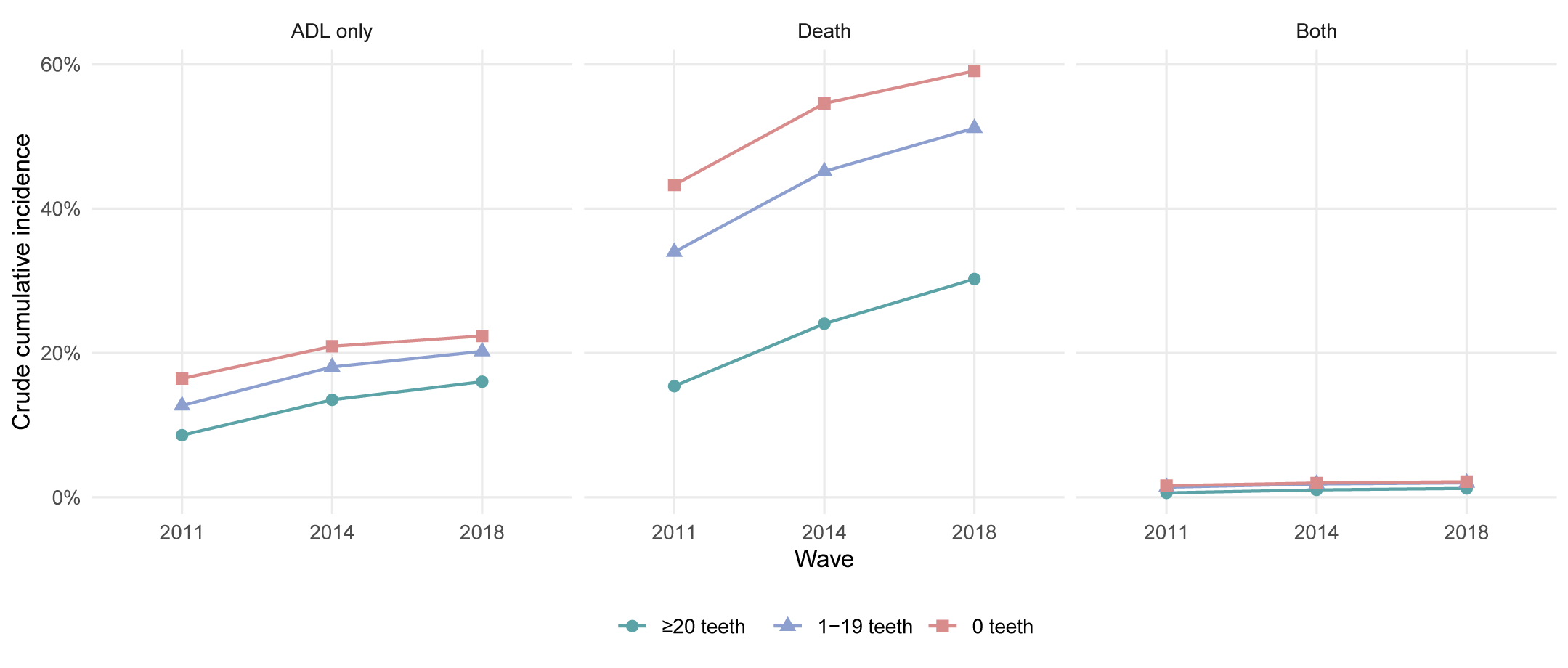

Supplement: Supplementary file 2 [file mmc2.zip › Figure S2.tif]

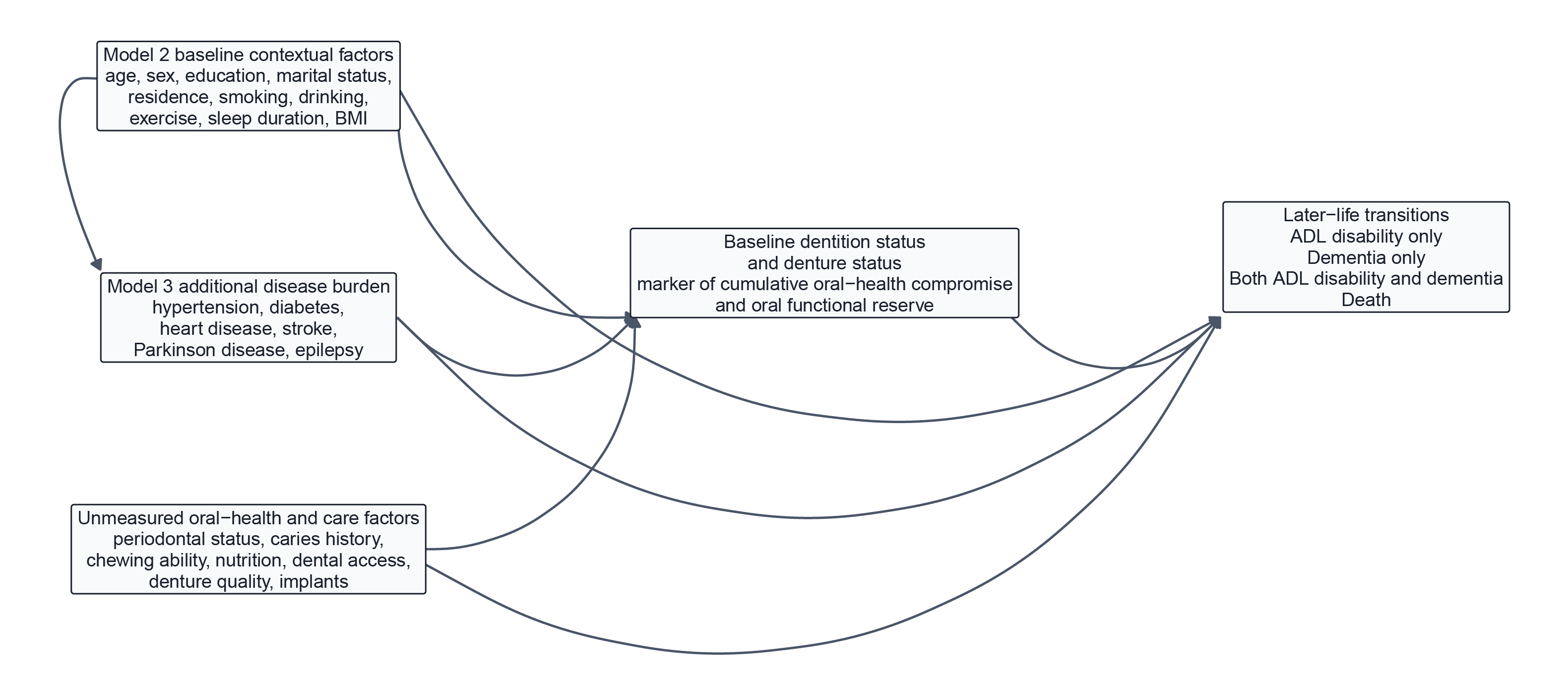

Supplement: Supplementary file 3 [file mmc3.zip › FigureS3.png]

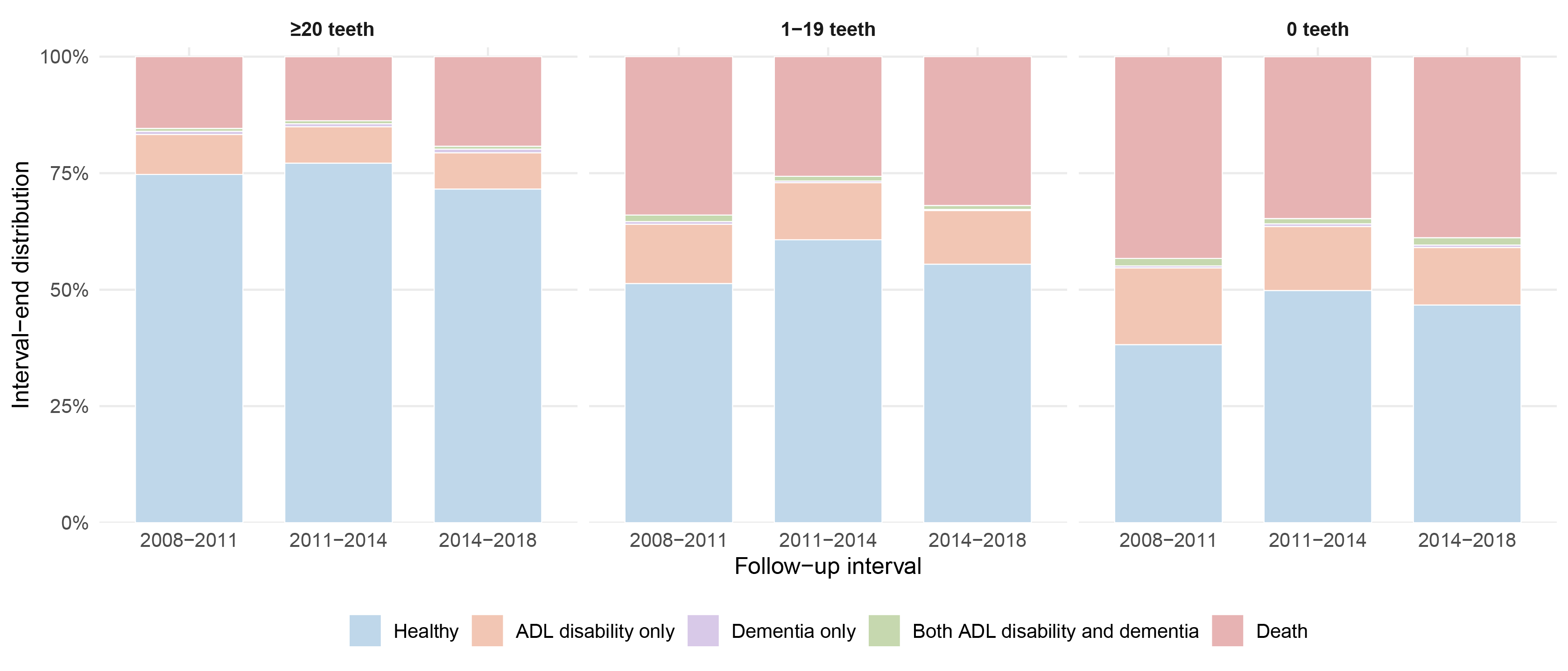

Supplement: Supplementary file 4 [file mmc4.zip › FigureS4.png]
