# Supplementary material for: Dentition Status and Denture Use in Relation to Later-Life Health Transitions in Older Chinese Adults
Source: Int Dent J. 2026 Jul 4;76(5):109710. doi: 10.1016/j.identj.2026.109710 (PMC13355603; doi:10.1016/j.identj.2026.109710)
Supplement: Supplementary file 5 [file mmc5.zip › Supplementary Appendix.docx]

**Supplementary Appendix**

**Supplementary Methods**

**eMethods 1. Analytic sample construction**

After verification and merging of required variables across study waves, participants were eligible if they were aged 65 years or older in 2008 and had valid baseline information on natural teeth count. The baseline healthy cohort was defined as participants with neither observed ADL disability nor reported dementia at baseline. The primary analytic cohort further required at least one usable follow-up interval under the prespecified sequential person-period rule. For the joint-exposure analysis, participants additionally required non-missing baseline denture information; no further exclusions occurred after restriction to the primary analytic cohort. The same primary analytic cohort was used for additional supplementary analyses unless otherwise specified.

**eMethods 2. Exposure definitions**

The primary exposure was baseline dentition status, categorized as at least 20 teeth, 1–19 teeth, and 0 teeth. In supplementary joint-exposure analyses, baseline dentition status was combined with baseline denture use to form six categories. The reference group was participants with at least 20 teeth and no dentures. An additional internal comparison of denture use versus no denture use was performed among participants with baseline dentition below 20 teeth. Baseline dentition and denture status were retained as the exposure definitions to preserve temporal ordering. Repeated tooth-count and denture status across follow-up waves were described in supplementary analyses, but these follow-up measures were not used to redefine the baseline exposures because they may reflect downstream changes after cohort entry.

**eMethods 3. Multi-state framework and cumulative outcome coding**

The primary analysis used a five-state framework consisting of Healthy, ADL disability only, Dementia only, Both ADL disability and dementia, and Death. ADL refers to activities of daily living. ADL disability was defined as a binary wave-specific indicator derived from six standard ADL items, with disability coded when at least one item indicated limitation or dependency and all six items were observed. The available CLHLS measure therefore captured the presence or absence of any ADL disability at each wave, rather than the number of impaired ADL items or disability severity. Dementia was defined using the CLHLS dementia item, and Death was identified from interval-specific mortality variables for 2008–2011, 2011–2014, and 2014–2018.

ADL disability and dementia were carried forward cumulatively once first observed, and Death was treated as an absorbing state. The allowed transitions were Healthy to ADL disability only, Healthy to Dementia only, Healthy to Both ADL disability and dementia, Healthy to Death, ADL disability only to Both ADL disability and dementia, ADL disability only to Death, Dementia only to Both ADL disability and dementia, Dementia only to Death, and Both ADL disability and dementia to Death. Because nonfatal states were assessed only at follow-up waves and Death was identified within intervals, transitions were classified according to interval-end observed states rather than exact within-interval event sequences.

Raw wave-to-wave reversals in ADL disability and dementia before carry-forward coding were tabulated separately to describe the extent of non-cumulative survey responses. These raw reversals were not used to redefine the primary states.

**eMethods 4. Person-period data and transition-specific models**

Person-period datasets were constructed for the three follow-up intervals: 2008–2011, 2011–2014, and 2014–2018. Participants contributed interval-specific observations only when both the start-of-interval and end-of-interval states were observable. When an interval was unusable because the end-of-interval state could not be determined, later intervals were not carried forward.

For each allowed transition, transition-specific risk sets were defined by retaining intervals that began in the relevant origin state. Event indicators were coded as 1 when the destination state of interest was reached by the end of the interval and 0 otherwise.

For descriptive interpretation, we also tabulated interval-end state distributions among person-intervals that started from the Healthy state. These descriptive distributions were calculated within each baseline dentition group and follow-up interval and were not model-adjusted.

**eMethods 5. Covariates, conceptual framework, and model specifications**

Baseline covariates included age, sex, education, marital status, residence, current smoking, current alcohol drinking, regular exercise, sleep duration, body mass index category, hypertension, diabetes, heart disease, stroke, Parkinson disease, and epilepsy. A prespecified conceptual framework was used to guide covariate selection and interpretation. Baseline dentition and denture status were interpreted as markers of cumulative oral-health compromise and oral functional reserve that may also reflect broader ageing-related vulnerability. Figure S3 summarizes how baseline contextual factors, systemic disease burden, unmeasured oral-health and care factors, and baseline oral status may relate to later-life transitions.

Three nested models were fitted. Model 1 adjusted for follow-up interval, age, and sex. Model 2 additionally adjusted for education, marital status, residence, smoking, drinking, exercise, sleep duration, and body mass index category and was designated as the principal model. Model 3 further adjusted for hypertension, diabetes, heart disease, stroke, Parkinson disease, and epilepsy and was interpreted as a supportive extended model because these conditions may reflect both baseline disease burden and pathways through which later-life vulnerability is expressed. Age was modelled using natural splines with 3 degrees of freedom, and robust standard errors were clustered at the participant level.

Categorical covariates were represented with explicit missing categories during model fitting to retain participants with otherwise usable exposure and outcome data. Baseline covariate missingness before missing-category coding was reported, and a complete-case sensitivity analysis was added for the Model 2 covariate set.

**eMethods 6. Supplementary analyses**

Several supplementary analyses were conducted to characterize sample selection, present the full five-state transition structure, assess robustness, and clarify key modelling and measurement decisions. These included an included-versus-excluded comparison using standardized mean differences to describe baseline imbalance rather than formal hypothesis testing [1]; full transition-specific results from the five-state Model 2 analysis; joint dentition-denture analyses for selected transitions from the healthy baseline state; a supportive three-state analysis using Healthy, Any impairment, and Death; a healthier restricted-sample analysis defined by further restricting the baseline healthy cohort to participants with no baseline hypertension, diabetes, heart disease, stroke, Parkinson disease, or epilepsy; age-stratified analyses with interaction testing; an internal denture comparison among participants with baseline dentition below 20 teeth; and descriptive crude cumulative-proportion plots for ADL disability only, Death, and Both ADL disability and dementia according to baseline dentition status. Because the healthier restricted sample required all six baseline conditions to be coded as No, participants with missing data for any of these conditions were not included in that analysis.

Additional analyses were conducted to clarify outcome coding, model specification, missing-data handling, and the interpretation of baseline oral-status measures. These analyses quantified raw wave-to-wave reversals in ADL disability and dementia before cumulative carry-forward coding, reported baseline covariate missingness before explicit missing-category modelling, documented the age-spline specification, summarized interval-end state distributions among intervals starting from Healthy, described repeated survey-reported tooth-count and denture status across follow-up waves, assessed complete-case sensitivity for the Model 2 covariate set, and explored whether baseline hypertension modified the association between baseline dentition status and the main Healthy-origin transitions.

**Supplementary Results**

**S1. Included versus excluded participants before primary analysis**

Baseline differences between participants included in and excluded from the primary analytic cohort are shown in Table S1. Excluded participants were older and generally had less favourable baseline characteristics than those retained, with the largest imbalance observed for age (standardized mean difference, 0.605). They were also more often female, unmarried, physically inactive, and edentulous at baseline. Overall, exclusion before entry into the primary analytic cohort appeared selective and tended to remove a frailer subset of participants.

**S2. Full transition-specific results in the five-state framework**

The complete set of transition-specific estimates from the five-state Model 2 analysis is presented in Table S2. The most informative results were concentrated in transitions from the baseline Healthy state, especially transitions to ADL disability only and Death. Dementia-related and other later transitions were sparse, and several estimates had wide confidence intervals or were not estimable. Dementia-specific transitions are therefore presented as exploratory estimates.

**S3.** **Joint dentition-denture analyses**

Table S3 and Figure S1 present the joint analyses of baseline dentition status and denture use for the two principal transitions from the healthy baseline state. Relative to the reference group of participants with at least 20 teeth and no dentures, the highest adjusted hazard of transition from Healthy to ADL disability only was observed among participants with 0 teeth and dentures. For Healthy to Death, the clearest excess hazard was seen among those with 0 teeth and no dentures, whereas the corresponding estimate for those with 0 teeth and dentures was notably weaker. Overall, denture status added prognostic separation within low-dentition categories, particularly for death. Because denture use may also reflect health status, dental-care access, treatment-seeking capacity, and selective survival, these joint-exposure findings were interpreted as descriptive risk stratification rather than as the isolated effect of denture use.

**S4. Supportive three-state analysis**

Results from the supportive three-state analysis are shown in Table S4. The simplified framework using Healthy, Any impairment, and Death yielded a pattern consistent with the primary analysis. In particular, baseline edentulism remained associated with higher risks of transition from Healthy to Any impairment and from Healthy to Death, indicating that the main findings were not dependent on the more detailed five-state structure.

**S5. Healthier restricted-sample analysis**

Table S5 shows the results from the healthier restricted-sample analysis, in which the baseline healthy cohort was further restricted to participants with no baseline hypertension, diabetes, heart disease, stroke, Parkinson disease, or epilepsy. The overall pattern remained similar to that of the main analysis. Baseline edentulism remained associated with higher risks of subsequent ADL disability only and death, whereas estimates for Healthy to Both ADL disability and dementia remained imprecise. This restricted-sample analysis showed a similar pattern after excluding participants with major chronic or neurological conditions at baseline.

**S6. Age-stratified analyses and interaction testing**

Age-stratified results are presented in Table S6. The associations of baseline edentulism with Healthy to ADL disability only and Healthy to Death were broadly similar in participants aged 65–79 years and those aged 80 years or older. Formal interaction testing did not indicate meaningful effect modification by age group. Estimates for Healthy to Both ADL disability and dementia were less stable because of the limited number of events.

**S7. Internal denture comparison among participants with baseline dentition below 20 teeth**

Table S7 summarizes the internal comparison of denture use versus no denture use among participants with baseline dentition below 20 teeth. Denture use was associated with lower subsequent death risk in this subgroup, whereas the corresponding association for Healthy to ADL disability only was weaker. The estimate for Healthy to Both ADL disability and dementia also favored denture use, although that transition was uncommon. Within the low-dentition subgroup, denture use appeared to distinguish mortality risk more clearly than progression to ADL disability only.

**S8. Descriptive cumulative-proportion patterns**

Figure S2 shows crude cumulative-proportion patterns for selected transitions from the healthy baseline state according to baseline dentition status. Separation across dentition groups was visible early and persisted through follow-up. By 2018, participants with 0 teeth had the highest crude cumulative proportions of both ADL disability only and Death, followed by those with 1–19 teeth, whereas the corresponding proportions were lowest among those with at least 20 teeth. The crude cumulative proportion of Both ADL disability and dementia remained low in all three groups.

**S9. Additional analyses on measurement, model specification, and robustness**

Additional analyses were performed to clarify outcome coding, baseline exposure interpretation, model specification, and missing-data handling. Raw wave-to-wave reversals before cumulative carry-forward coding are shown in Table S8. ADL disability reversals were observed mainly in later intervals, whereas dementia reversals were uncommon. Baseline covariate missingness in the primary analytic cohort was low overall and is reported in Table S9. The age distribution and natural-spline specification used in regression models are documented in Table S10.

Table S11 and Figure S4 present interval-end health-state distributions among person-intervals that started from the Healthy state, stratified by baseline dentition status. Death and ADL disability accounted for most observed non-Healthy transitions, whereas Dementia only and Both ADL disability and dementia remained uncommon. Repeated survey-reported tooth-count and denture status across follow-up waves are presented in Tables S12 and S13, providing descriptive context for the use of baseline dentition and denture status as exposure measures.

Complete-case sensitivity analyses for the Model 2 covariate set are shown in Table S14. The complete-case estimates were consistent with the main Model 2 estimates for the principal Healthy-origin transitions. Exploratory interaction tests between baseline dentition status and hypertension are shown in Table S15; no clear interaction pattern was observed.

References

1. Austin PC. Balance diagnostics for comparing the distribution of baseline covariates between treatment groups in propensity-score matched samples. Statistics in medicine. 2009;28(25):3083-3107.

**Supplementary Tables**

**Table S1. Baseline characteristics of participants included in and excluded from the primary analytic sample, presented with standardized mean differences.**

| **Characteristic** | **Included primary sample** | **Excluded before primary sample** | **SMD** |
| --- | --- | --- | --- |
| N | 10,186 | 6,335 |  |
| **Age, years** | 84.97 (10.97) | 91.55 (10.78) | 0.605 |
| **Female sex** | 5436 (53.4) | 4121 (65.1) | 0.239 |
| **Education, years** |  |  | 0.186 |
| 0 | 6099 (59.9) | 4348 (68.6) |  |
| 1-6 | 3034 (29.8) | 1436 (22.7) |  |
| ≥7 | 1049 (10.3) | 550 (8.7) |  |
| Missing | 4 (0.0) | 1 (0.0) |  |
| **Married** | 3649 (35.8) | 1252 (19.8) | 0.364 |
| **City/Town residence** | 3545 (34.8) | 3018 (47.6) | 0.263 |
| **Current smoking** | 2085 (20.5) | 720 (11.4) | 0.251 |
| **Current drinking** | 2017 (19.8) | 761 (12.0) | 0.214 |
| **Regular exercise** |  |  | 0.211 |
| No | 7042 (69.1) | 4961 (78.3) |  |
| Yes | 3144 (30.9) | 1373 (21.7) |  |
| Missing | 0 (0.0) | 1 (0.0) |  |
| **Sleep duration** |  |  | 0.206 |
| <7 h | 2610 (25.6) | 1721 (27.2) |  |
| 7-9 h | 4889 (48.0) | 2466 (38.9) |  |
| >9 h | 2661 (26.1) | 2096 (33.1) |  |
| Missing | 26 (0.3) | 52 (0.8) |  |
| **Body mass index, kg/m²** |  |  | 0.298 |
| <18.5 | 3101 (30.4) | 2235 (35.3) |  |
| 18.5-23.9 | 5556 (54.5) | 3056 (48.2) |  |
| 24.0-27.9 | 1166 (11.4) | 557 (8.8) |  |
| ≥28 | 276 (2.7) | 150 (2.4) |  |
| Missing | 87 (0.9) | 337 (5.3) |  |
| **Denture use** |  |  | 0.038 |
| No | 7347 (72.1) | 4663 (73.6) |  |
| Yes | 2839 (27.9) | 1671 (26.4) |  |
| Missing | 0 (0.0) | 1 (0.0) |  |
| **Hypertension** |  |  | 0.132 |
| No | 8070 (79.2) | 4896 (77.3) |  |
| Yes | 1974 (19.4) | 1223 (19.3) |  |
| Missing | 142 (1.4) | 216 (3.4) |  |
| **Diabetes** |  |  | 0.182 |
| No | 9855 (96.8) | 5918 (93.4) |  |
| Yes | 231 (2.3) | 189 (3.0) |  |
| Missing | 100 (1.0) | 228 (3.6) |  |
| **Heart disease** |  |  | 0.192 |
| No | 9278 (91.1) | 5475 (86.4) |  |
| Yes | 824 (8.1) | 655 (10.3) |  |
| Missing | 84 (0.8) | 205 (3.2) |  |
| **Stroke** |  |  | 0.276 |
| No | 9689 (95.1) | 5555 (87.7) |  |
| Yes | 423 (4.2) | 574 (9.1) |  |
| Missing | 74 (0.7) | 206 (3.3) |  |
| **Baseline dentition status** |  |  | 0.307 |
| ≥20 teeth | 1954 (19.2) | 688 (10.9) |  |
| 1-19 teeth | 4945 (48.5) | 2797 (44.2) |  |
| 0 teeth | 3287 (32.3) | 2850 (45.0) |  |

Standardized mean differences are presented to describe baseline imbalance between participants retained in the primary analytic sample and those excluded before primary analysis. Because this table is descriptive rather than inferential, P values are intentionally omitted. Age is presented as mean (SD); other variables are shown as n (%).

**Table S2. Full transition-specific associations between baseline dentition status and subsequent multi-state progression in the primary five-state model (Model 2).**

| **Transition** | **Comparison** | **Events** | **HR (95% CI)** | **P value** |
| --- | --- | --- | --- | --- |
| **Healthy to ADL disability only** | | | | |
|  | 1-19 teeth vs ≥20 teeth | 2048 | 1.08 (0.94-1.24) | 0.275 |
|  | 0 teeth vs ≥20 teeth | 2048 | 1.20 (1.04-1.39) | 0.015 |
| **Healthy to Dementia only** | | | | |
|  | 1-19 teeth vs ≥20 teeth | 85 | 0.54 (0.31-0.94) | 0.028 |
|  | 0 teeth vs ≥20 teeth | 85 | 0.58 (0.32-1.07) | 0.081 |
| **Healthy to Both ADL disability and dementia** | | | | |
|  | 1-19 teeth vs ≥20 teeth | 194 | 1.13 (0.71-1.79) | 0.613 |
|  | 0 teeth vs ≥20 teeth | 194 | 1.14 (0.69-1.87) | 0.606 |
| **Healthy to Death** | | | | |
|  | 1-19 teeth vs ≥20 teeth | 5063 | 1.10 (1.00-1.21) | 0.048 |
|  | 0 teeth vs ≥20 teeth | 5063 | 1.18 (1.06-1.31) | 0.002 |
| **ADL disability only to Both ADL disability and dementia** | | | | |
|  | 1-19 teeth vs ≥20 teeth | 44 | 1.03 (0.40-2.66) | 0.952 |
|  | 0 teeth vs ≥20 teeth | 44 | 0.96 (0.33-2.80) | 0.943 |
| **ADL disability only to Death** | | | | |
|  | 1-19 teeth vs ≥20 teeth | 1147 | 1.16 (0.92-1.46) | 0.206 |
|  | 0 teeth vs ≥20 teeth | 1147 | 1.14 (0.90-1.46) | 0.276 |
| **Dementia only to Both ADL disability and dementia** | | | | |
|  | 1-19 teeth vs ≥20 teeth | 18 | NE |  |
|  | 0 teeth vs ≥20 teeth | 18 | NE |  |
| **Dementia only to Death** | | | | |
|  | 1-19 teeth vs ≥20 teeth | 32 | NE |  |
|  | 0 teeth vs ≥20 teeth | 32 | NE |  |
| **Both ADL disability and dementia to Death** | | | | |
|  | 1-19 teeth vs ≥20 teeth | 169 | 0.79 (0.44-1.42) | 0.436 |
|  | 0 teeth vs ≥20 teeth | 169 | 1.02 (0.59-1.77) | 0.947 |

NE indicates not estimable. Dementia-related and other sparse transitions are retained for completeness but should be interpreted as exploratory rather than stable findings.

**Table S3. Joint exposure analyses of baseline dentition status and denture use for the Healthy to ADL disability only and Healthy to Death transitions.**

| **Transition** | **Comparison** | **Events** | **Model 1 HR (95% CI)** | **Model 2 HR (95% CI)** | **Model 3 HR (95% CI)** |
| --- | --- | --- | --- | --- | --- |
| **Healthy to ADL disability only** | | | | | |
|  | ≥20 teeth, denture vs ≥20 teeth, no denture | 2048 | 1.32 (0.99-1.78) | 1.22 (0.91-1.64) | 1.22 (0.91-1.63) |
|  | 1-19 teeth, no denture vs ≥20 teeth, no denture | 2048 | 1.02 (0.88-1.18) | 1.08 (0.93-1.25) | 1.08 (0.93-1.26) |
|  | 1-19 teeth, denture vs ≥20 teeth, no denture | 2048 | 1.17 (0.98-1.40) | 1.17 (0.97-1.40) | 1.15 (0.96-1.38) |
|  | 0 teeth, no denture vs ≥20 teeth, no denture | 2048 | 1.06 (0.89-1.27) | 1.13 (0.94-1.35) | 1.12 (0.94-1.34) |
|  | 0 teeth, denture vs ≥20 teeth, no denture | 2048 | 1.29 (1.10-1.52) | 1.30 (1.10-1.53) | 1.28 (1.08-1.51) |
| **Healthy to Death** | | | | | |
|  | ≥20 teeth, denture vs ≥20 teeth, no denture | 5063 | 0.81 (0.63-1.05) | 0.87 (0.67-1.13) | 0.87 (0.67-1.13) |
|  | 1-19 teeth, no denture vs ≥20 teeth, no denture | 5063 | 1.23 (1.11-1.37) | 1.17 (1.06-1.29) | 1.17 (1.05-1.29) |
|  | 1-19 teeth, denture vs ≥20 teeth, no denture | 5063 | 0.87 (0.76-0.99) | 0.88 (0.76-1.00) | 0.87 (0.76-1.00) |
|  | 0 teeth, no denture vs ≥20 teeth, no denture | 5063 | 1.45 (1.29-1.62) | 1.33 (1.18-1.49) | 1.33 (1.18-1.49) |
|  | 0 teeth, denture vs ≥20 teeth, no denture | 5063 | 1.07 (0.96-1.20) | 1.06 (0.94-1.19) | 1.05 (0.94-1.18) |

The reference group is ≥20 teeth without denture use. Results are shown with the three model specifications in adjacent columns to avoid repeated row blocks.

**Table S4. Supportive three-state analyses using Healthy, Any impairment, and Death as the state structure (Model 2).**

| **Transition** | **Comparison** | **Events** | **HR (95% CI)** | **P value** |
| --- | --- | --- | --- | --- |
| **Healthy to Any impairment** | | | | |
|  | 1-19 teeth vs ≥20 teeth | 2327 | 1.04 (0.92-1.18) | 0.514 |
|  | 0 teeth vs ≥20 teeth | 2327 | 1.15 (1.01-1.32) | 0.042 |
| **Healthy to Death** | | | | |
|  | 1-19 teeth vs ≥20 teeth | 5063 | 1.10 (1.00-1.21) | 0.048 |
|  | 0 teeth vs ≥20 teeth | 5063 | 1.18 (1.06-1.31) | 0.002 |
| **Any impairment to Death** | | | | |
|  | 1-19 teeth vs ≥20 teeth | 1348 | 1.18 (0.96-1.45) | 0.118 |
|  | 0 teeth vs ≥20 teeth | 1348 | 1.17 (0.94-1.45) | 0.161 |

Any impairment indicates the occurrence of ADL disability and/or dementia. This supportive analysis was performed to provide a more stable summary of progression when dementia-specific transitions were sparse in the five-state framework.

**Table S5. Healthier restricted-sample analyses for selected transitions from the healthy baseline state.**

| **Transition** | **Comparison** | **Events** | **Model 1 HR (95% CI)** | **Model 2 HR (95% CI)** | **Model 3 HR (95% CI)** |
| --- | --- | --- | --- | --- | --- |
| **Healthy to ADL disability only** | | | | | |
|  | 0 teeth vs ≥20 teeth | 1362 | 1.22 (1.02-1.48) | 1.27 (1.05-1.54) | NE |
|  | 1-19 teeth vs ≥20 teeth | 1362 | 1.10 (0.92-1.31) | 1.16 (0.97-1.38) | NE |
| **Healthy to Both ADL disability and dementia** | | | | | |
|  | 0 teeth vs ≥20 teeth | 125 | 1.32 (0.68-2.58) | 1.35 (0.69-2.63) | NE |
|  | 1-19 teeth vs ≥20 teeth | 125 | 1.32 (0.69-2.49) | 1.37 (0.73-2.57) | NE |
| **Healthy to Death** | | | | | |
|  | 0 teeth vs ≥20 teeth | 3703 | 1.26 (1.11-1.43) | 1.19 (1.05-1.35) | NE |
|  | 1-19 teeth vs ≥20 teeth | 3703 | 1.17 (1.04-1.32) | 1.11 (0.99-1.25) | NE |

The healthier restricted sample was defined by further restricting the baseline healthy cohort to participants with no baseline hypertension, diabetes, heart disease, stroke, Parkinson disease, or epilepsy. Participants with missing data for any of these conditions were not included. Model 3 was not estimable in this restricted sample because the additional disease covariates were fixed by the restriction criteria. Results are shown with Model 1, Model 2, and Model 3 presented in adjacent columns. NE indicates not estimable.

**Table S6. Age-stratified associations and interaction tests for selected transitions from the healthy baseline state.**

| **Transition** | **Age group** | **Comparison** | **Events** | **HR (95% CI)** | **P value** | **P for interaction** |
| --- | --- | --- | --- | --- | --- | --- |
| **Healthy to ADL disability only** | | | | | | |
|  | 65-79 | 1-19 teeth vs ≥20 teeth | 489 | 1.12 (0.92-1.37) | 0.255 | 0.671 |
|  | 65-79 | 0 teeth vs ≥20 teeth | 489 | 1.15 (0.88-1.51) | 0.31 |  |
|  | 80+ | 1-19 teeth vs ≥20 teeth | 1559 | 1.05 (0.87-1.27) | 0.594 |  |
|  | 80+ | 0 teeth vs ≥20 teeth | 1559 | 1.19 (0.98-1.44) | 0.077 |  |
| **Healthy to Death** | | | | | | |
|  | 65-79 | 1-19 teeth vs ≥20 teeth | 812 | 1.16 (0.99-1.36) | 0.067 | 0.937 |
|  | 65-79 | 0 teeth vs ≥20 teeth | 812 | 1.27 (1.03-1.56) | 0.025 |  |
|  | 80+ | 1-19 teeth vs ≥20 teeth | 4251 | 1.08 (0.95-1.22) | 0.224 |  |
|  | 80+ | 0 teeth vs ≥20 teeth | 4251 | 1.15 (1.02-1.31) | 0.027 |  |
| **Healthy to Both ADL disability and dementia** | | | | | | |
|  | 65-79 | 1-19 teeth vs ≥20 teeth | 36 | 1.40 (0.68-2.87) | 0.361 | 0.375 |
|  | 65-79 | 0 teeth vs ≥20 teeth | 36 | 0.87 (0.30-2.52) | 0.801 |  |
|  | 80+ | 1-19 teeth vs ≥20 teeth | 158 | 0.96 (0.54-1.69) | 0.883 |  |
|  | 80+ | 0 teeth vs ≥20 teeth | 158 | 1.03 (0.57-1.84) | 0.93 |  |

Age-stratified results are shown for participants aged 65-79 years and 80 years or older. Interaction P values test effect modification by age stratum within each transition.

**Table S7. Internal comparison of denture use within participants with baseline dentition below 20 teeth.**

| **Transition** | **Comparison** | **Events** | **Model 1 HR (95% CI)** | **Model 2 HR (95% CI)** | **Model 3 HR (95% CI)** |
| --- | --- | --- | --- | --- | --- |
| **Healthy to ADL disability only** | | | | | |
|  | Denture vs no denture within baseline <20 teeth | 1735 | 1.19 (1.06-1.32) | 1.12 (1.00-1.26) | 1.10 (0.99-1.24) |
| **Healthy to Both ADL disability and dementia** | | | | | |
|  | Denture vs no denture within baseline <20 teeth | 170 | 0.71 (0.48-1.04) | 0.64 (0.43-0.96) | 0.62 (0.41-0.92) |
| **Healthy to Death** | | | | | |
|  | Denture vs no denture within baseline <20 teeth | 4472 | 0.72 (0.67-0.78) | 0.77 (0.72-0.83) | 0.77 (0.72-0.83) |

The comparison evaluates denture use versus no denture use among participants with baseline dentition below 20 teeth. Results are displayed side by side across the three model specifications to avoid repeated row blocks.

**Table S8. Raw wave-to-wave reversals in ADL disability and dementia before cumulative carry-forward coding.**

| **Domain** | **Interval** | **Observed pairs** | **Raw reversal, n (%)** | **Raw incident, n (%)** | **Stable impaired, n** | **Stable unimpaired, n** |
| --- | --- | --- | --- | --- | --- | --- |
| ADL disability | 2008-2011 | 7113 | 0 (0.00) | 1561 (21.95) | 0 | 5552 |
|  | 2011-2014 | 4397 | 192 (4.37) | 653 (14.85) | 376 | 3176 |
|  | 2014-2018 | 2047 | 79 (3.86) | 337 (16.46) | 115 | 1516 |
| Dementia | 2008-2011 | 6973 | 0 (0.00) | 194 (2.78) | 0 | 6779 |
|  | 2011-2014 | 4131 | 29 (0.70) | 102 (2.47) | 13 | 3987 |
|  | 2014-2018 | 1832 | 11 (0.60) | 43 (2.35) | 6 | 1772 |

The table is based on the baseline healthy cohort. A raw reversal denotes a change from impaired at the previous wave to unimpaired at the subsequent wave before cumulative carry-forward coding. Percentages are calculated among participants with observed paired domain measurements for the corresponding interval. ADL, activities of daily living.

**Table S9. Baseline missingness before explicit missing-category coding in the primary analytic cohort.**

| **Variable** | **N** | **Missing, n** | **Missing, %** |
| --- | --- | --- | --- |
| Baseline natural tooth count | 10,186 | 0 | 0.00 |
| Baseline denture use | 10,186 | 0 | 0.00 |
| Baseline ADL disability | 10,186 | 0 | 0.00 |
| Baseline dementia | 10,186 | 0 | 0.00 |
| Age | 10,186 | 0 | 0.00 |
| Sex | 10,186 | 0 | 0.00 |
| Education | 10,186 | 4 | 0.04 |
| Marital status | 10,186 | 0 | 0.00 |
| Residence | 10,186 | 0 | 0.00 |
| Current smoking | 10,186 | 0 | 0.00 |
| Current alcohol drinking | 10,186 | 0 | 0.00 |
| Regular exercise | 10,186 | 0 | 0.00 |
| Sleep duration | 10,186 | 26 | 0.26 |
| Body mass index category | 10,186 | 87 | 0.85 |
| Hypertension | 10,186 | 142 | 1.39 |
| Diabetes | 10,186 | 100 | 0.98 |
| Heart disease | 10,186 | 84 | 0.82 |
| Stroke | 10,186 | 74 | 0.73 |
| Parkinson disease | 10,186 | 211 | 2.07 |
| Epilepsy | 10,186 | 13 | 0.13 |

The primary analytic cohort included 10,186 participants. Missingness was assessed before categorical covariates were represented with explicit missing categories during model fitting. ADL, activities of daily living.

**Table S10. Age distribution and natural spline specification used in regression models.**

| **Item** | **Value** |
| --- | --- |
| Analytic cohort, n | 10,186 |
| Minimum age, years | 65.00 |
| 25th percentile, years | 76.00 |
| Median age, years | 86.00 |
| 75th percentile, years | 93.00 |
| Maximum age, years | 116.00 |
| Spline specification | splines::ns(age, df = 3) |
| Degrees of freedom | 3 |
| Internal knots, years | 80; 91 |
| Boundary knots, years | 65; 116 |

Age was modelled using a natural cubic spline with 3 degrees of freedom. Internal and boundary knots were generated from the age distribution in the primary analytic cohort.

**Table S11. Interval-end health states among intervals starting from Healthy, by baseline dentition status.**

| **Interval** | **Baseline dentition** | **Risk set, n** | **Healthy, n (%)** | **ADL disability only, n (%)** | **Dementia only, n (%)** | **Both ADL disability and dementia, n (%)** | **Death, n (%)** |
| --- | --- | --- | --- | --- | --- | --- | --- |
| 2008-2011 | ≥20 teeth | 1954 | 1460 (74.72) | 168 (8.60) | 13 (0.67) | 12 (0.61) | 301 (15.40) |
|  | 1-19 teeth | 4945 | 2538 (51.32) | 629 (12.72) | 28 (0.57) | 69 (1.40) | 1681 (33.99) |
|  | 0 teeth | 3287 | 1256 (38.21) | 541 (16.46) | 14 (0.43) | 53 (1.61) | 1423 (43.29) |
| 2011-2014 | ≥20 teeth | 1230 | 949 (77.15) | 96 (7.80) | 8 (0.65) | 8 (0.65) | 169 (13.74) |
|  | 1-19 teeth | 2150 | 1305 (60.70) | 264 (12.28) | 7 (0.33) | 22 (1.02) | 552 (25.67) |
|  | 0 teeth | 1068 | 532 (49.81) | 147 (13.76) | 6 (0.56) | 12 (1.12) | 371 (34.74) |
| 2014-2018 | ≥20 teeth | 630 | 451 (71.59) | 49 (7.78) | 5 (0.79) | 4 (0.63) | 121 (19.21) |
|  | 1-19 teeth | 930 | 516 (55.48) | 107 (11.51) | 2 (0.22) | 8 (0.86) | 297 (31.94) |
|  | 0 teeth | 381 | 178 (46.72) | 47 (12.34) | 2 (0.52) | 6 (1.57) | 148 (38.85) |

Percentages are calculated within each baseline dentition group and follow-up interval among person-intervals that started from the Healthy state. This descriptive table presents interval-end distributions and is not model-adjusted. Blank cells in grouping columns indicate the same value as the nearest non-empty entry above. ADL, activities of daily living.

**Table S12. Repeated survey-reported natural tooth-count status across waves in the primary analytic cohort.**

| **Baseline dentition** | **Wave** | **Cohort N** | **Current ≥20 teeth, n (%)** | **Current 1-19 teeth, n (%)** | **Current 0 teeth, n (%)** | **Missing/not observed, n (%)** |
| --- | --- | --- | --- | --- | --- | --- |
| ≥20 teeth | 2008 | 1954 | 1954 (100.00) | 0 (0.00) | 0 (0.00) | 0 (0.00) |
|  | 2011 |  | 961 (49.18) | 571 (29.22) | 103 (5.27) | 319 (16.33) |
|  | 2014 |  | 633 (32.40) | 500 (25.59) | 105 (5.37) | 716 (36.64) |
|  | 2018 |  | 300 (15.35) | 328 (16.79) | 77 (3.94) | 1249 (63.92) |
| 1-19 teeth | 2008 | 4945 | 0 (0.00) | 4945 (100.00) | 0 (0.00) | 0 (0.00) |
|  | 2011 |  | 394 (7.97) | 2129 (43.05) | 708 (14.32) | 1714 (34.66) |
|  | 2014 |  | 237 (4.79) | 1296 (26.21) | 538 (10.88) | 2874 (58.12) |
|  | 2018 |  | 89 (1.80) | 588 (11.89) | 284 (5.74) | 3984 (80.57) |
| 0 teeth | 2008 | 3287 | 0 (0.00) | 0 (0.00) | 3287 (100.00) | 0 (0.00) |
|  | 2011 |  | 74 (2.25) | 399 (12.14) | 1365 (41.53) | 1449 (44.08) |
|  | 2014 |  | 53 (1.61) | 184 (5.60) | 721 (21.93) | 2329 (70.85) |
|  | 2018 |  | 20 (0.61) | 74 (2.25) | 272 (8.28) | 2921 (88.87) |

Baseline dentition status was the exposure used in the main analysis. Follow-up tooth-count categories are shown descriptively to indicate how survey-reported oral status changed or became unobserved across waves. Blank cells in grouping columns indicate the same value as the nearest non-empty entry above.

**Table S13. Repeated survey-reported denture status across waves in the primary analytic cohort.**

| **Baseline dentition** | **Baseline denture status** | **Wave** | **Cohort N** | **Current denture, n (%)** | **Current no denture, n (%)** | **Missing/not observed, n (%)** |
| --- | --- | --- | --- | --- | --- | --- |
| ≥20 teeth | No denture at baseline | 2008 | 1674 | 0 (0.00) | 1674 (100.00) | 0 (0.00) |
|  |  | 2011 |  | 297 (17.74) | 1105 (66.01) | 272 (16.25) |
|  |  | 2014 |  | 260 (15.53) | 800 (47.79) | 614 (36.68) |
|  |  | 2018 |  | 169 (10.10) | 443 (26.46) | 1062 (63.44) |
|  | Denture at baseline | 2008 | 280 | 280 (100.00) | 0 (0.00) | 0 (0.00) |
|  |  | 2011 |  | 138 (49.29) | 107 (38.21) | 35 (12.50) |
|  |  | 2014 |  | 109 (38.93) | 82 (29.29) | 89 (31.79) |
|  |  | 2018 |  | 67 (23.93) | 42 (15.00) | 171 (61.07) |
| 1-19 teeth | No denture at baseline | 2008 | 3941 | 0 (0.00) | 3941 (100.00) | 0 (0.00) |
|  |  | 2011 |  | 428 (10.86) | 2019 (51.23) | 1494 (37.91) |
|  |  | 2014 |  | 305 (7.74) | 1191 (30.22) | 2445 (62.04) |
|  |  | 2018 |  | 167 (4.24) | 506 (12.84) | 3268 (82.92) |
|  | Denture at baseline | 2008 | 1004 | 1004 (100.00) | 0 (0.00) | 0 (0.00) |
|  |  | 2011 |  | 569 (56.67) | 247 (24.60) | 188 (18.73) |
|  |  | 2014 |  | 402 (40.04) | 182 (18.13) | 420 (41.83) |
|  |  | 2018 |  | 214 (21.31) | 85 (8.47) | 705 (70.22) |
| 0 teeth | No denture at baseline | 2008 | 1732 | 0 (0.00) | 1732 (100.00) | 0 (0.00) |
|  |  | 2011 |  | 97 (5.60) | 701 (40.47) | 934 (53.93) |
|  |  | 2014 |  | 53 (3.06) | 290 (16.74) | 1389 (80.20) |
|  |  | 2018 |  | 20 (1.15) | 78 (4.50) | 1634 (94.34) |
|  | Denture at baseline | 2008 | 1555 | 1555 (100.00) | 0 (0.00) | 0 (0.00) |
|  |  | 2011 |  | 841 (54.08) | 221 (14.21) | 493 (31.70) |
|  |  | 2014 |  | 499 (32.09) | 134 (8.62) | 922 (59.29) |
|  |  | 2018 |  | 215 (13.83) | 59 (3.79) | 1281 (82.38) |

Baseline denture status was used in the joint-exposure and internal denture analyses. Follow-up denture status is shown descriptively and does not redefine the baseline exposure. Blank cells in grouping columns indicate the same value as the nearest non-empty entry above.

**Table S14. Complete-case sensitivity analysis for Model 2 covariates.**

| **Transition** | **Risk set, n** | **Events, n** | **Comparison** | **HR (95% CI)** | **P value** |
| --- | --- | --- | --- | --- | --- |
| Healthy to ADL disability only | 16,434 | 2017 | 1-19 teeth vs ≥20 teeth | 1.08 (0.94-1.24) | 0.272 |
|  |  |  | 0 teeth vs ≥20 teeth | 1.20 (1.04-1.39) | 0.015 |
| Healthy to Both ADL disability and dementia |  | 193 | 1-19 teeth vs ≥20 teeth | 1.13 (0.71-1.79) | 0.609 |
|  |  |  | 0 teeth vs ≥20 teeth | 1.13 (0.69-1.86) | 0.623 |
| Healthy to Death |  | 4998 | 1-19 teeth vs ≥20 teeth | 1.10 (1.00-1.21) | 0.055 |
|  |  |  | 0 teeth vs ≥20 teeth | 1.18 (1.07-1.31) | 0.001 |

Estimates were obtained from transition-specific discrete-time complementary log-log models with cluster-robust standard errors. Model 2 adjusted for follow-up interval, age, sex, education, marital status, residence, smoking, drinking, exercise, sleep duration, and body mass index category. Complete-case analysis excluded intervals with missing values in Model 2 covariates. CI, confidence interval; HR, hazard ratio.

**Table S15. Exploratory interaction tests between baseline dentition status and hypertension.**

| **Transition** | **Risk set, n** | **Events, n** | **Interaction χ²** | **df** | **P value** |
| --- | --- | --- | --- | --- | --- |
| Healthy to ADL disability only | 16,339 | 2027 | 2.612 | 2 | 0.271 |
| Healthy to Both ADL disability and dementia | 16,339 | 193 | 0.519 | 2 | 0.772 |
| Healthy to Death | 16,339 | 4991 | 0.305 | 2 | 0.859 |

nteraction tests evaluated cross-product terms between baseline dentition status and baseline hypertension in transition-specific Model 2 specifications. These analyses were exploratory and were not used to modify the main interpretation. df, degrees of freedom.

**Supplementary Figure Legends**

**
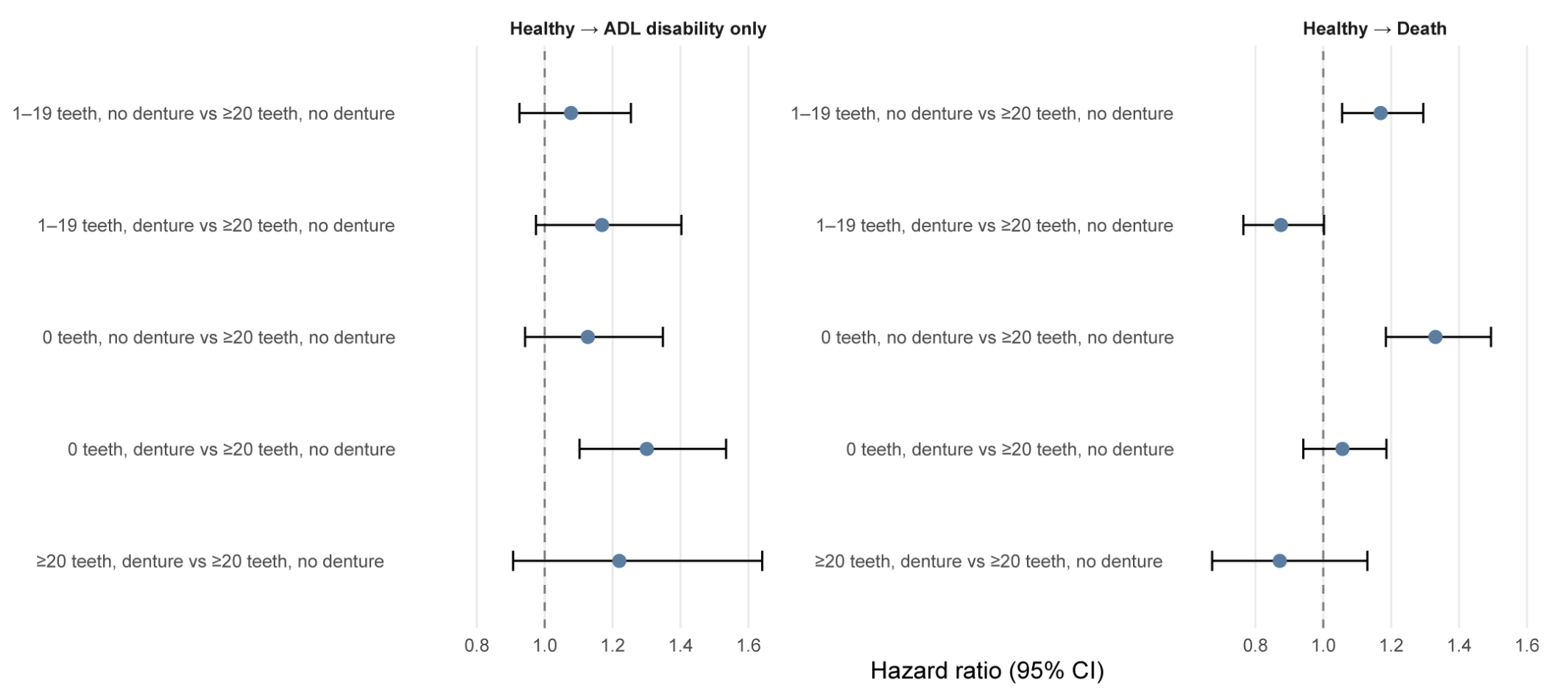
**

**Figure S1. Joint associations of baseline dentition status and denture use with subsequent transitions from Healthy to ADL disability only and from Healthy to Death.** Hazard ratios and 95% confidence intervals were estimated from Model 2. The reference group was participants with at least 20 teeth and no denture use at baseline. Model 2 adjusted for follow-up interval, age modelled with a natural spline with 3 degrees of freedom, sex, education, marital status, residence, current smoking, current alcohol drinking, regular exercise, sleep duration, and body mass index category. All participants were without observed ADL disability or reported dementia at baseline.


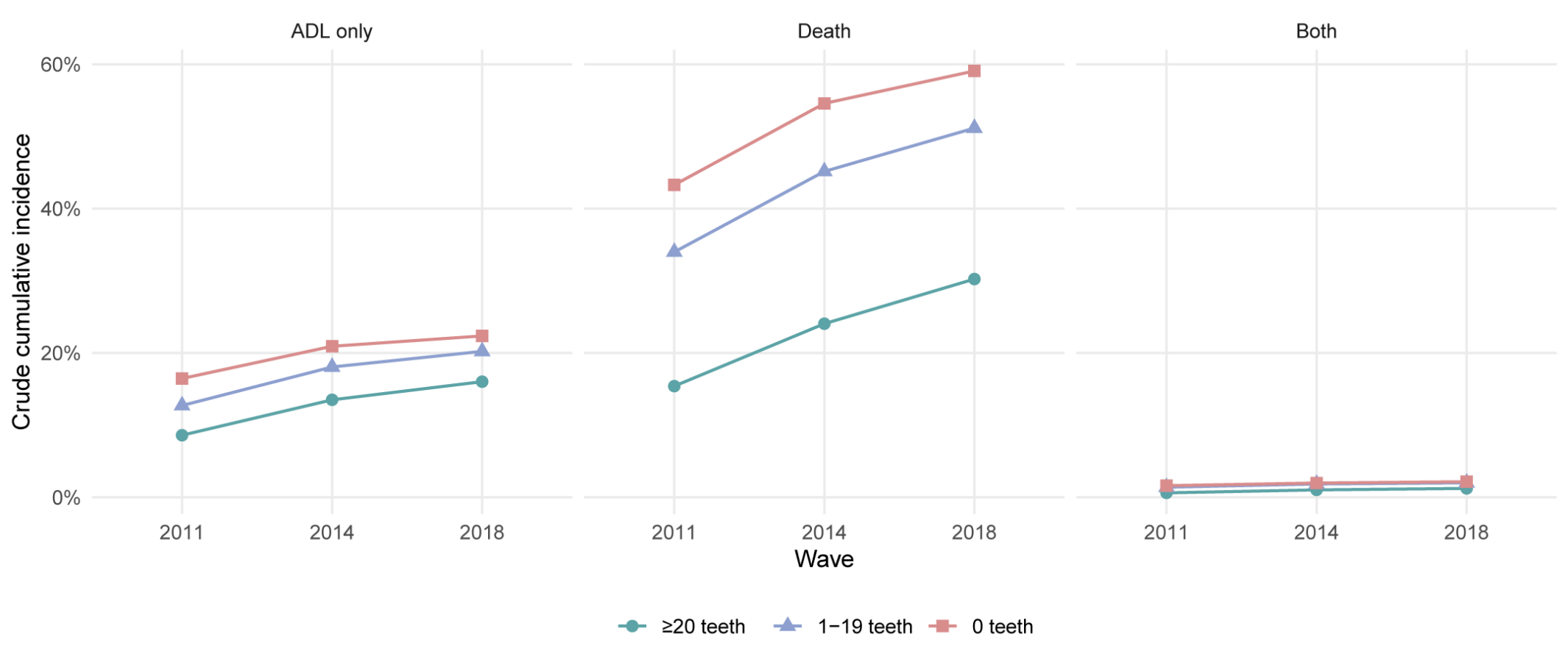


**Figure S2. Crude cumulative proportions of selected transitions from the healthy baseline state according to baseline dentition status across follow-up waves.** Curves show the observed crude cumulative proportions of ADL disability only, Death, and Both ADL disability and dementia at the 2011, 2014, and 2018 follow-up waves according to baseline dentition status. Estimates are descriptive and unadjusted. All participants were without observed ADL disability or reported dementia at baseline.


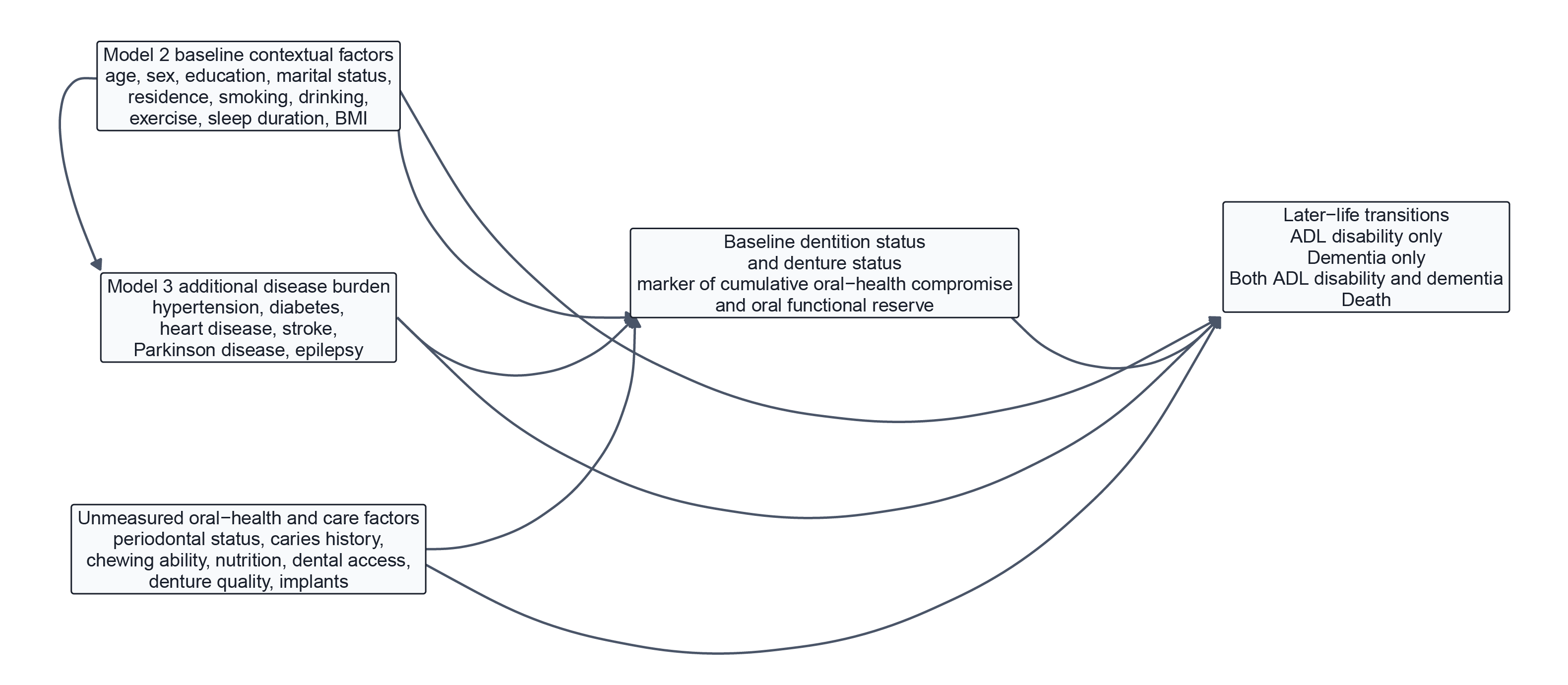
 **Figure S3. Conceptual framework for interpreting baseline dentition status, covariate adjustment, and later-life transitions.** Baseline dentition and denture status were interpreted as markers of cumulative oral-health compromise and oral functional reserve. Model 2 adjusted for baseline contextual factors, whereas Model 3 additionally included major disease-burden variables and was interpreted as a supportive extended model. Unmeasured oral-health and care factors may influence both baseline oral status and later-life transitions. ADL, activities of daily living; BMI, body mass index.

**
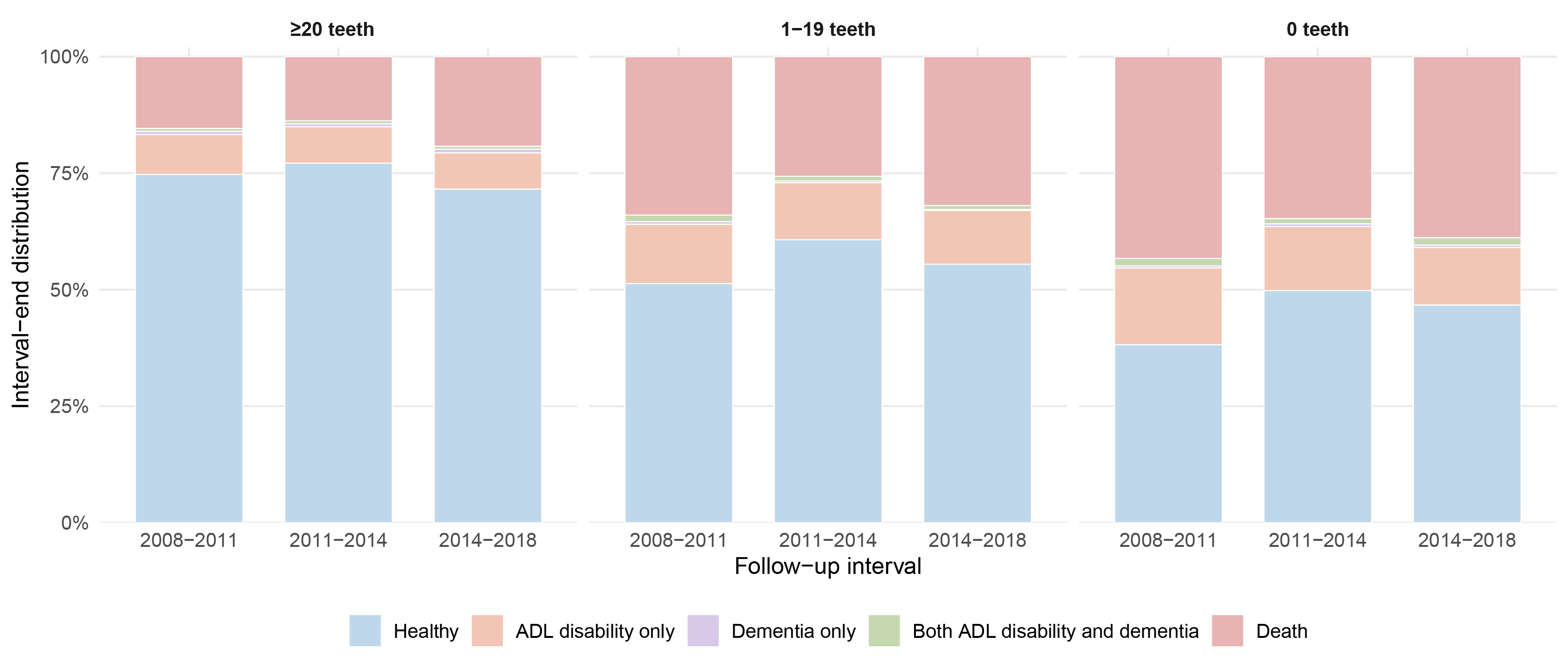
 Figure S4. Interval-end health-state distribution among intervals starting from Healthy, by baseline dentition status.** Bars show the distribution of end-of-interval states among person-intervals that started from the Healthy state. Percentages were calculated within each baseline dentition group and follow-up interval. This figure presents descriptive interval-end distributions and is not model-adjusted. ADL, activities of daily living.
